# Anomalous oxygen concentration increases in benthic experiments from the Clarion Clipperton Zone are not related to polymetallic nodules

**DOI:** 10.12688/f1000research.181486.1

**Published:** 2026-05-14

**Authors:** Alexander P. Webber, Patrick Downes, Joaquim Bento, Leigh Marsh, Felipe S. Freitas, Michael Clarke

**Affiliations:** 1 Environmental, The Metals Company, Vancouver, BC, V6E 2J3, Canada

**Keywords:** Polymetallic nodules, dark oxygen, image segmentation, deep sea mining, clarion clipperton zone

## Abstract

**Background:**

Polymetallic nodules in the deep sea of the Clarion Clipperton zone (CCZ) have been implicated in the abiotic production of oxygen during benthic chamber respirometer experiments. The hypothesis, termed “dark oxygen”, was supported with a correlation between average nodule surface area and oxygen production rates, given as a Spearman’s correlation coefficient of *⍴* = 0.664, *p* = 0.031, n = 11, suggesting that larger polymetallic nodules cause more oxygen production. However, this correlation and the dark oxygen hypothesis appears to be incompatible with the observation that experiments in the western CCZ and a control experiment recorded increasing oxygen with no nodules present.

**Methods:**

To investigate the dark oxygen hypothesis, we repeated the surface area analysis using image segmentation, and expanded the dataset with three additional chamber images, the weight of the nodules recovered from the chambers and a further three experiments for which nodule weight data are available.

**Results:**

We find no significant correlations between total nodule area, average area, nodule weight or average nodule weight and rising oxygen concentrations, indicating that there is no link between the abundance or size of polymetallic nodules and rising oxygen concentrations. The inclusion of the three additional chamber images in the analysis reduces the significance of the previously stated correlation to *⍴* = 0.433, *p* = 0.122, with confidence intervals that cross zero (−0.13, 0.78). Furthermore, we note that a link between polymetallic nodules and rising oxygen concentrations is directly contradicted by the experiments from the western CCZ and the control experiment that recorded rising oxygen when no polymetallic nodules were present.

**Conclusion:**

Since all these experiments recorded increasing oxygen concentration regardless of the size, abundance, or presence of polymetallic nodules, we find that increasing oxygen concentrations in these experiments is unrelated to polymetallic nodules.

## Introduction

Polymetallic nodules, which are manganese- and iron-oxide concretions found in certain parts of the seafloor, have been linked to the potential production of oxygen in benthic chamber lander experiments by Sweetman et al. (2024). ^1^ These experiments, conducted in the Clarion Clipperton Zone (CCZ), recorded a net increase of dissolved oxygen instead of the expected oxygen consumption that results from benthic respiration, which has been consistently measured by various other studies in similar settings with polymetallic nodules present. ^2– 6^ As such, the results and conclusions of Sweetman et al. (2024) are anomalous in the context of scientific consensus on the topic, and the work has attracted criticism from the scientific community, detailing many methodological concerns and issues relating to experimental design, reporting and interpretation of the data. ^7– 13^ In Sweetman et al. (2024), the rising oxygen concentrations were attributed to the polymetallic nodules, and this hypothesis, termed “dark oxygen”, was supported with a statistically significant but low sample size correlation between average nodule surface area and the amount of oxygen produced ( *⍴* = 0.664, *p* = 0.031, n = 11), with the implication being that larger nodules correspond to more oxygen production. However, Sweetman et al. (2024) failed to highlight that the total nodule surface area in each chamber shows no correlation with oxygen production, which might be expected if they were producing oxygen. Furthermore, Sweetman et al. (2024) fail to report that chambers from the western CCZ that recorded increasing oxygen did not contain any nodules, ^14, 15^ and fail to report a control experiment which also recorded increasing oxygen concentration without nodules. ^7^ These observations preclude the possibility that polymetallic nodules have any role in oxygen concentration increases in those experiments, and are incompatible with the stated correlation with nodule surface area. This suggests that the correlation between nodule surface area and oxygen production may be spurious, leading to the need for further investigation.

Here, we present a re-analysis of the nodule surface areas, and expand the dataset by including three additional chambers that were omitted from the original analysis of Sweetman et al. (2024). We publish the photos that were used to estimate nodule surface area, and outline a method using image segmentation to extract 2D surface area values. We also report the weight of nodules that were recovered from the chambers, which is a direct measurement of the size and abundance of nodules present during the incubations. Contrary to the finding in Sweetman et al. (2024), we find no significant correlation between oxygen concentration increases and average nodule surface area. We also find no significant correlation between total nodule surface area, nodule weight or average nodule weight and oxygen concentration increases. We therefore reject the claim that the magnitude of oxygen concentration increase is related to the size or abundance of polymetallic nodules present. These findings are consistent with the chambers and control experiment without nodules, which indicate that oxygen increased regardless of whether nodules were present or not. We therefore conclude that there is no link between polymetallic nodules and the observed rises in oxygen concentration in the benthic chamber respirometer experiments.

## Methods

Benthic chamber respirometer experiments were conducted on Nauru Ocean Resources Inc. (NORI) research cruises 5D, 5E and 7A to the NORI-D contract area in the eastern CCZ. ^1^ These took place in April to June 2021, November to December 2021 and August to September 2022, respectively. The experiments used a KUM benthic chamber respirometer lander system comprising three incubation chambers (KUM, Kiel). Details of the benthic chamber respirometer deployments, oxygen optode data and experimental procedures are described fully in Sweetman et al. (2024); here we summarise only those aspects relevant to the nodule surface area re-analysis. Photographs were taken of the top surface of benthic chambers after recovery, showing the seafloor with nodules sitting on the sediment surface. After photographs were taken of the chambers, the sediment, fauna and nodules in the chambers were processed.

The chamber photographs were not intended to be used for quantitative analysis and consequently are not ideally composed for the task of image segmentation. They are taken at an oblique angle, often with poor lighting, with undesirable shadows and highlights that make segmentation difficult. Additionally, some photos were taken with water or fogging present on the camera lens. Nevertheless, the photographs can be improved by correcting the geometry and enhancing the contrast between nodules and the sediment. The workflow for image preparation is outlined as follows:

1. Use image processing software to bring the four corners of the chamber box to squareness.
2. Crop the image to the four corners of the box, where the sediment surface meets the edges of the box. The image should now be proportional to the dimensions of the chamber, which is 22x22 cm.
3. Adjust the brightness and contrast of the image, if necessary, to ensure the best colour separation between the nodules and the surrounding sediment.
4. Use the paint tools to remove artifacts on the nodules, such as reflection highlights and mobilised sediment that has washed onto the nodule surface during sampling.

**Figure 1.**
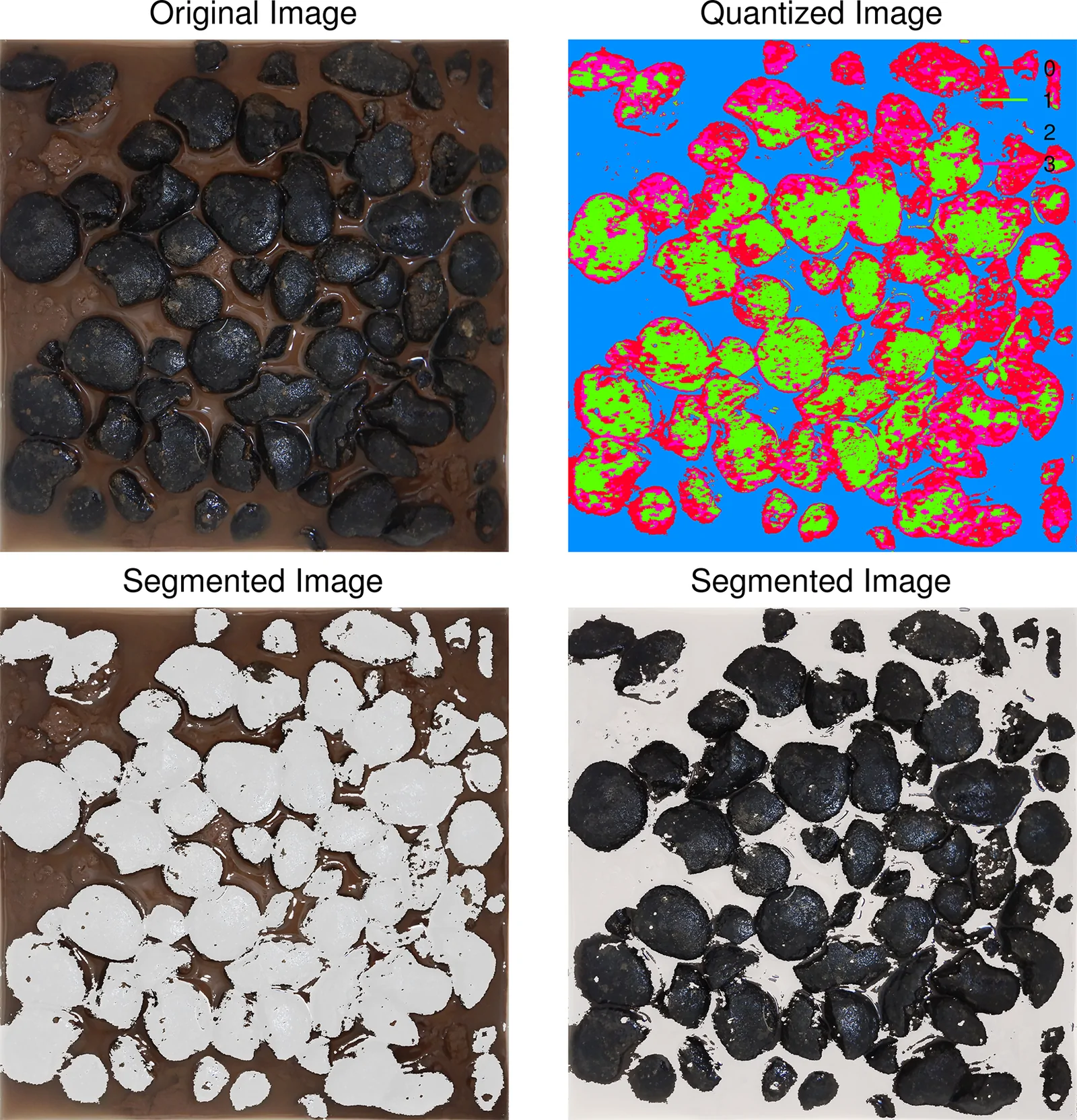
Example segmentation using AKS282 chamber 3, which is one of the images with reasonable quality. K-means clustering is able to pick out the difference between nodules and sediment with four clusters in this case. Additionally, the nodules are clean of sediment and don’t require any editing.

In some cases, steps three and four were applied iteratively to optimise segmentation performance, and some images required extensive editing to outline the nodules. After image preparation, the photos were accessed using Python, and image segmentation was applied with k-means clustering in HSV colour space, implemented using the Scikit-learn library. ^16^ The number of clusters was tuned for each image to best represent the nodules, and the nodule clusters were assigned. Alternative, more sophisticated segmentation algorithms were explored, but the variation in image quality resulted in the best outcome using k-means clustering, which is easily tuned to each image. Pixels classified as nodules were counted, and the known chamber dimensions (22 x 22 cm) were used to convert pixel counts to area, yielding the 2D nodule-covered area for each image ( Figure 1). Average nodule surface area was obtained by dividing the nodule surface area by the number of nodules in the image, following Sweetman et al. (2024).

**Figure 2.**
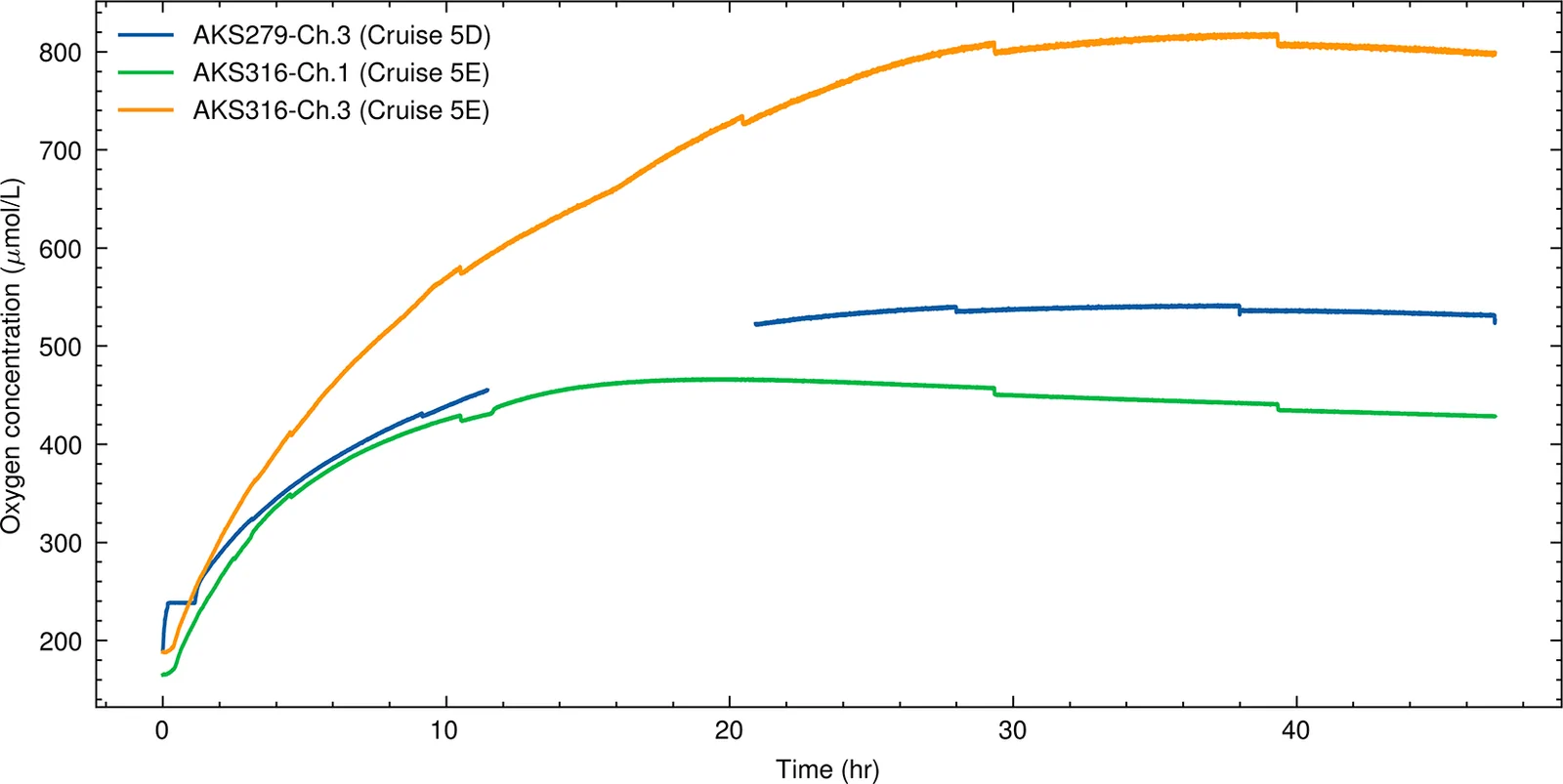
The three additional chambers, which were published in Sweetman et al. (2024) but not included in the surface area analysis, record the full incubation period and the maximum oxygen concentration reached during that period.

In our analysis we include three additional chambers for which photographs and oxygen data are available: AKS279 chamber 3 and AKS316 chamber 1 and chamber 3. Although these were reported as showing increases in oxygen concentration, and the optode data and total oxygen production were published and used to support the hypothesis of oxygen production, ^1^ they were not included in the surface area analysis of Sweetman et al. (2024). A gap in the optode data for AKS279 chamber 3 was noted, but this does not appear to exclude the part of the timeseries where the oxygen concentration reaches a maximum ( Figure 2). AKS316 chamber 1 and chamber 3 record optode data for the full 47 hour period. We can see no reason to exclude these chambers from the analysis; the top-shot photographs are present, nodule weight and water volume data are available, and there are no comments in the sample logs regarding chamber failure.

We publish data for the weight of nodules collected from the benthic chambers after recovery. The nodules were collected and the wet weight was recorded from two depth intervals, the uppermost (0–2 cm) layer and the lower (2–5 cm) layer. Total nodule weight is obtained by adding these together and average nodule weight is obtained by dividing the weight of nodules in the 0–2 cm layer, which represents the nodules visible in the photograph, by the number of nodules counted in the photograph. These data are available for 17 chambers with corresponding oxygen concentration data, including three chambers for which photographs are not available. We therefore have a sample size of n = 17 for nodule weight analysis, compared to n = 14 for the nodule surface area analysis. Nodule weight and oxygen data is available for AKS331 chamber 2, but this was excluded from the analysis due to an incomplete oxygen optode data timeseries. AKS282 chamber 3 was also excluded from the analysis due to missing the 0–2 cm nodule weight value, which was not recorded in the offshore logs, despite the chamber photograph showing many nodules on the sediment surface.

We also report oxygen concentration increases from three benthic chamber lander deployments (AKS254, AKS257 and AKS261) from cruise KM1808 of the R/V Kilo Moana in 2018. ^14^ Although O _2_ flux and total O _2_ production in these chambers was not previously published, we have used available data ^17^ and the same method applied by Sweetman et al. (2024); taking the difference between the T _0_ O _2_ concentration and maximum O _2_ concentration reached during the experiment, and using the calculated volume of water above the level of the sediment in the chamber to reach a total moles of O _2_ that has apparently been produced during the experiment. The average nodule area, total nodule area and weight of nodules are all zero for these experiments, since no nodules were observed. ^14, 15^

Correlation coefficients and confidence intervals were calculated using the Scipy ^17^ and Pingouin libraries. ^18^ Oxygen optode data, production rates and total oxygen production are available from Sweetman et al. (2024). ^1^ See *Data Availability* for underlying data, metadata and python code used in this study.

## Results

**Figure 3.**
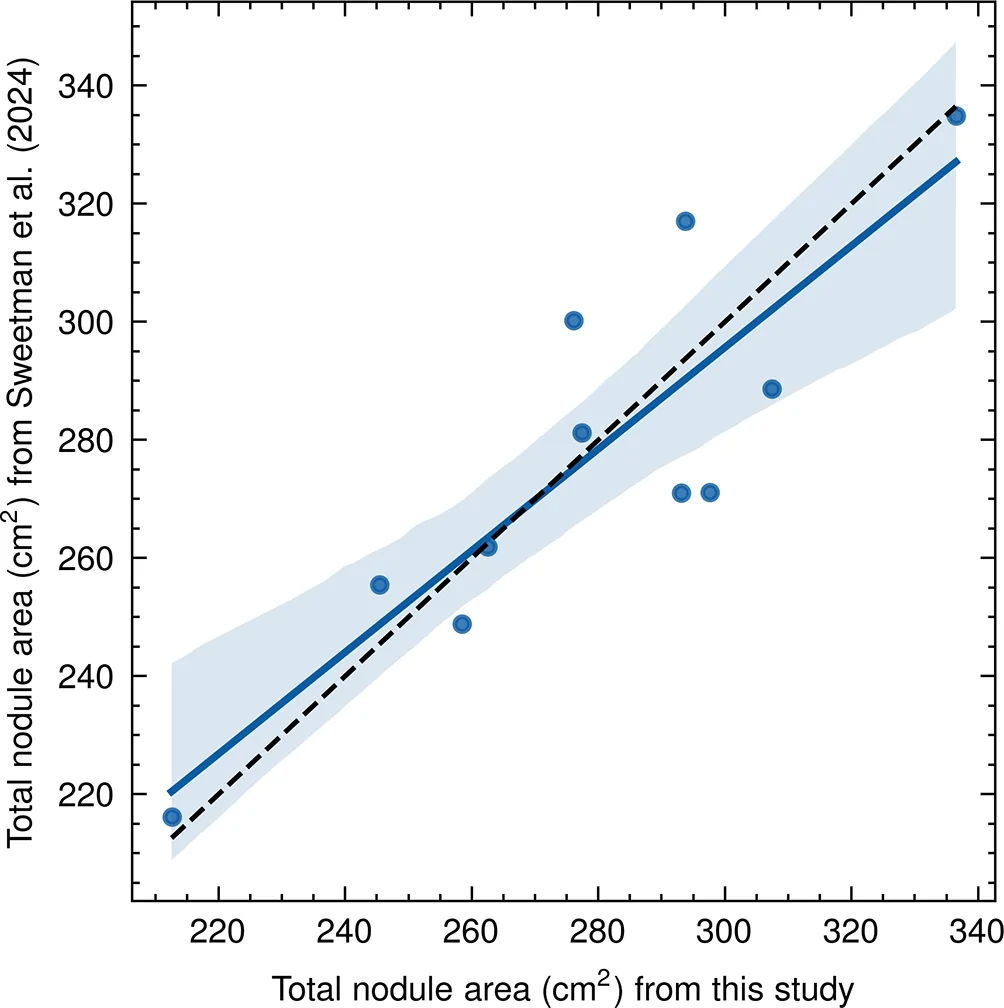
Comparison between the nodule surface areas published in Sweetman et al. (2024) and those determined in this study. Blue line and shading are the regression analysis with 95% confidence intervals, the black dashed line is the 1:1 correspondence line.

The nodule surface area was found to vary between 212.6 cm ^2^ and 336.5 cm ^2^ ( Table 1). When compared to the previously published values, ^1^ there is good agreement ( Figure 3). Of the chambers which are present in both studies, no chamber shows greater than 9.8% difference in total nodule surface area, and there is good linear correlation with a near 1:1 relationship, indicating no systematic difference in the way in which the surface areas were arrived at.

**Table 1.** Oxygen production and flux rates, as published in Sweetman et al. (2024), ^1^ along with nodule weight data, nodule surface areas determined in this study, and additional flux rates derived from data in. ^1, 14^ Table notes correspond as follows: 1: Surface area analysis not published in Sweetman et al. (2024), 2: Chamber failed to collect sediment or nodules, 3: No T0 O _2_ reading but initial O _2_ concentration is within the range of other chambers, 4: No T0 O _2_ reading but the O _2_ flux was published in Sweetman et al. (2024), 5: O _2_ flux calculated following procedure of Sweetman et al. (2024), 6: Appears to be missing the initial O _2_ rise - excluded from analysis, 7: Weight of nodules in 0–2 cm layer not recorded.

Chamber	Incubation Time (h)	T0 O _2_ (μmol)	Max O _2_ (μmol)	ΔO _2_ max (μmol)	End O _2_ (μmol)	ΔO _2_ end (μmol)	Water Volume (L)	Flux (mmol O _2_ m ^−2^)	Total O _2_ production (μmol)	Layer 0-2 cm nodule weight (g)	Layer 2-5 cm nodule weight (g)	Total nodule weight (g)	Nodule area (cm ^2^)	Number of nodules	Average nodule area (cm ^2^)	Average nodule weight (g)	Notes
AKS254_Ch3	36.6	88.71	157.02	68.30	156.84	68.13	6.29	5.81	429.77	0	0	0	0	0			5
AKS257_Ch3	38	85.99	176.86	90.87	176.61	90.63	8.47	10.02	769.69	0	0	0	0	0			5
AKS261_Ch2	39	86.06	216.91	130.84	213.75	127.68				0	0	0	0	0			2
AKS261_Ch3	39	79.82	143.39	63.57	140.42	60.60	4.52	3.48	287.18	0	0	0	0	0			5
AKS268_Ch3	47	200.43	464.78	264.34	460.61	260.18	3.81	10.46	1007.68	656	126	782.0	297.65	50	5.95	13.12	
AKS273_Ch3	47	192.47	528.44	335.97	523.49	331.02	4.60	16.06	1544.78	535	118	653.0	212.58	23	9.24	23.26	
AKS276_Ch3	47	196.36	366.94	170.58	344.04	147.67	3.93	6.13	670.87	336	54	390.0	258.49	50	5.17	6.72	
AKS279_Ch3	47	190.22	542.08	351.86	526.16	335.94	4.66	16.51	1639.31	363	46.55	409.6	260.77	41	6.36	8.85	1
AKS282_Ch3	47	189.40	242.80	53.40	224.86	35.46	4.60	1.72	245.53		15.5		293.14	68	4.31		7
AKS286_Ch3	47	186.71	241.02	54.31	225.08	38.36	6.05	2.45	328.58	454	11.99	466.0	262.48	53	4.95	8.57	
AKS313_Ch1	47	165.42	318.77	153.34	318.16	152.74											2
AKS313_Ch2	47	188.14	300.89	112.76	282.56	94.42											2
AKS316_Ch1	47	164.96	466.70	301.74	428.65	263.68	2.12	5.89	638.93	512	0	512.0	291.11	46	6.33	11.13	1
AKS316_Ch3	47	188.12	818.65	630.53	798.46	610.34	2.42	15.58	1525.89	662	0	662.0	254.71	57	4.47	11.61	1
AKS318_Ch1	47	161.00	150.80	-10.21	150.86	-10.15											2
AKS318_Ch3	46.8	183.96	308.51	124.55	308.40	124.44	2.78	3.67	346.62	596	0	596.0					4
AKS321_Ch1	47	161.69	146.53	-15.16	146.68	-15.01											2
AKS321_Ch3	45.7	192.09	217.88	25.79	194.54	2.45	2.78	0.07	71.77	651	212	863.0					3
AKS328_Ch2	47	162.83	232.58	69.74	214.72	51.88	3.69	2.02	257.38	620	9.7	629.7	336.49	75	4.49	8.27	4
AKS328_Ch3	47	183.33	362.55	179.22	341.49	158.16	3.81	6.36	683.08	539	17.39	556.4	277.43	78	3.56	6.91	
AKS329_Ch1	47	195.59	429.12	233.53	428.94	233.34	3.87	9.53	904.23	628	127	755.0					
AKS329_Ch2	47	200.47	400.25	199.77	398.85	198.38	3.87	8.10	773.52	658	46	704.0	276.09	49	5.63	13.43	
AKS331_Ch2	46.5	200.11	222.13	22.02	195.01	-5.11	2.72	-0.14	59.95	666	38	704.0	256.67	55	4.67	12.11	6
AKS331_Ch3	47	202.89	247.46	44.57	216.59	13.70	3.21	0.46	142.91	574	34	608.0					
AKS334_Ch2	47	196.65	606.32	409.67	605.71	409.06	4.17	18.02	1710.16	626	41	667.0	293.73	58	5.06	10.79	
AKS334_Ch3	47	206.08	332.86	126.78	310.51	104.43	5.14	5.67	651.96	601	20	621.0	307.46	67	4.59	8.97	
AKS336_Ch3	47	201.22	515.82	314.60	505.16	303.94	4.48	14.36	1408.47	607	15	622.0	245.40	52	4.72	11.67	

**Figure 4.**
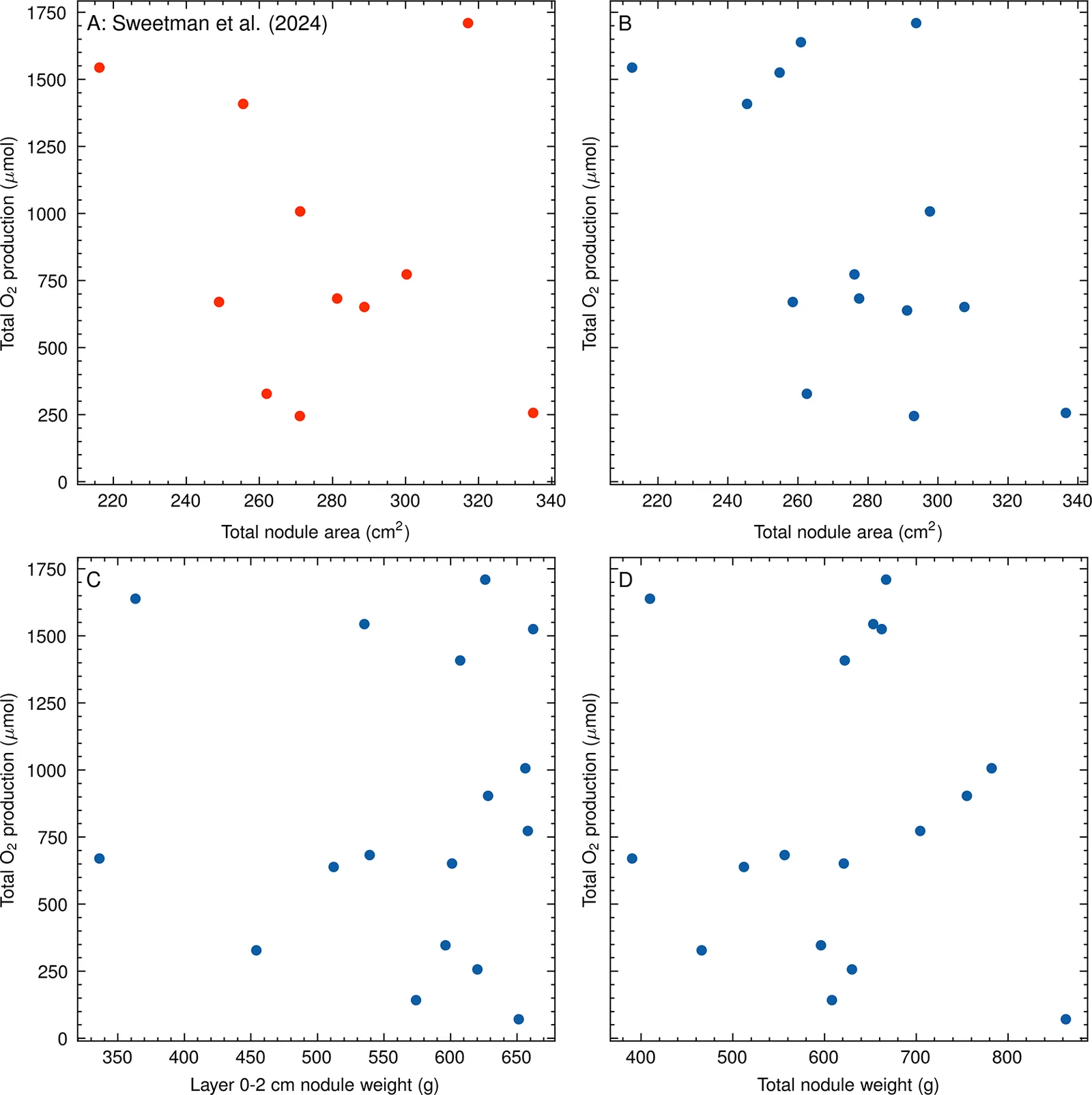
Data from Sweetman et al. (2024) and results of this study. A: Total nodule area from Sweetman et al. (2024) vs oxygen production shows no correlation. B: Total nodule area from this study vs oxygen production shows no correlation. C and D: Nodule weight in the upper layer and total nodule weight, respectively, vs oxygen production. Both show no correlation.

**Figure 5.**
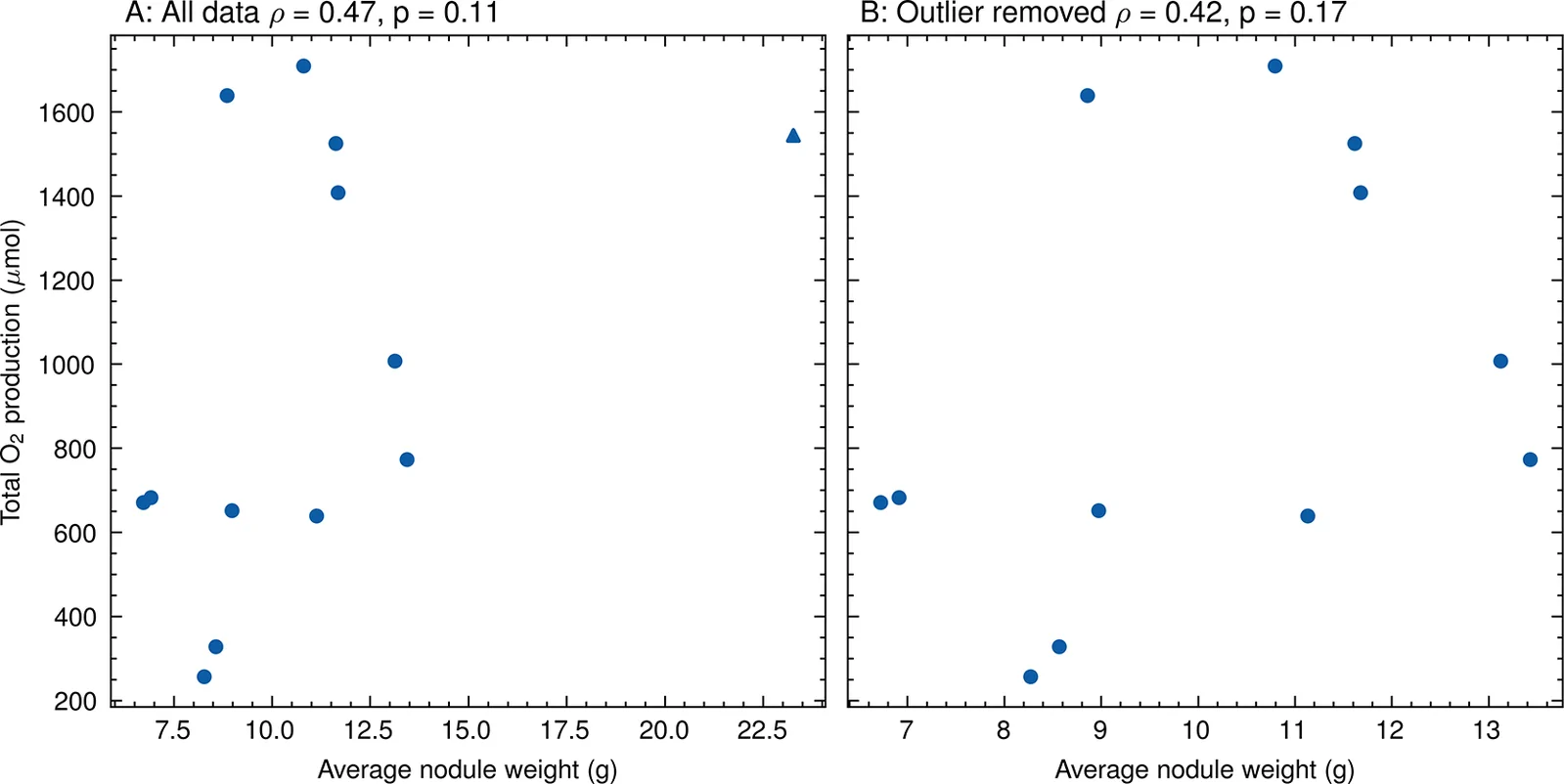
Average nodule weight vs total oxygen production. A: All data including the outlier value at 23.3 g (triangle symbol). B: Without the outlier value. No significant correlation is produced in either case.

**Figure 6.**
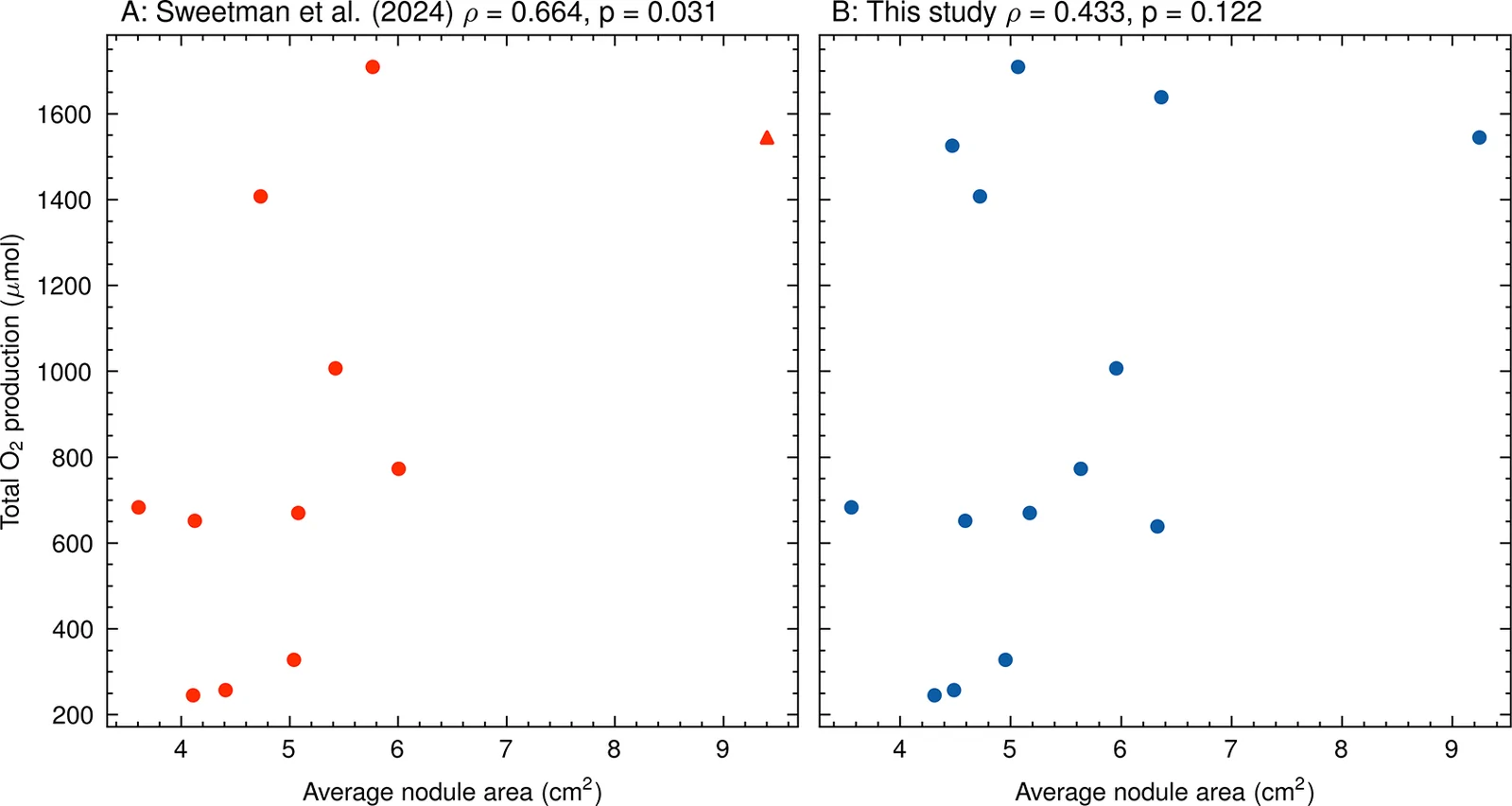
Comparison of the correlation between average nodule area and oxygen production. A: Data from Sweetman et al. (2024). The data point shown as a triangle symbol is a univariate outlier, defined by median absolute deviation. ^19^ When this outlier is excluded from their dataset, the correlation is insignificant (Spearman’s ⍴ = 0.5758, p = 0.0816, 95% CI = −0.08, 0.88) B: Data from this study. The correlation apparent in A is not statistically significant when the complete dataset is included in the analysis.

We find no correlation between total nodule surface area and oxygen production (Spearman’s *⍴ =* −0.451, p = 0.106) ( Figure 4A, B) and no correlation between nodule weight in the upper layer (0–2 cm) of the chamber and oxygen production (Spearman’s *⍴ =* 0.115, p = 0.660), nor total nodule weight and oxygen production (Spearman’s *⍴ =* 0.145, p = 0.58) ( Figure 4C, D). Average nodule weight also does not show any correlation (Spearman’s *⍴ =* 0.47, p = 0.11, or *⍴ =* 0.42, p = 0.17 with an outlier value removed) ( Figure 5). In contrast to the published correlation in Sweetman et al. (2024), we do not observe a statistically significant correlation between average nodule surface area and oxygen production ( Figure 6). Instead, we calculate a Spearman’s correlation of *⍴* = 0.433, *p* = 0.122, with 95% confidence intervals that cross zero (−0.13, 0.78). Therefore, these data do not corroborate the finding that oxygen concentration increases in the chambers are correlated to average nodule surface area. We note that our correlation becomes significant (Spearman’s *⍴ =* 0.609, p = 0.047) when excluding AKS279 chamber 3 and AKS316 chamber 1 and chamber 3, which were omitted from the analysis in Sweetman et al. (2024). Furthermore, AKS273 chamber 3 is a univariate outlier in the dataset of Sweetman et al. (2024), defined by median absolute deviation ^19^ ( Figure 6A). When this outlier is excluded from their dataset, the correlation becomes insignificant (Spearman’s *⍴* = 0.5758, *p* = 0.0816, 95% CI = −0.08, 0.88). In addition, the chambers from deployments AKS254, AKS257 and AKS261 in the western CCZ recorded oxygen increases that are well within the range of the rest of the data (429.8, 769.7 and 287.2 μmol O _2_) with no polymetallic nodules present ( Table 1).

## Discussion

**Figure 7.**
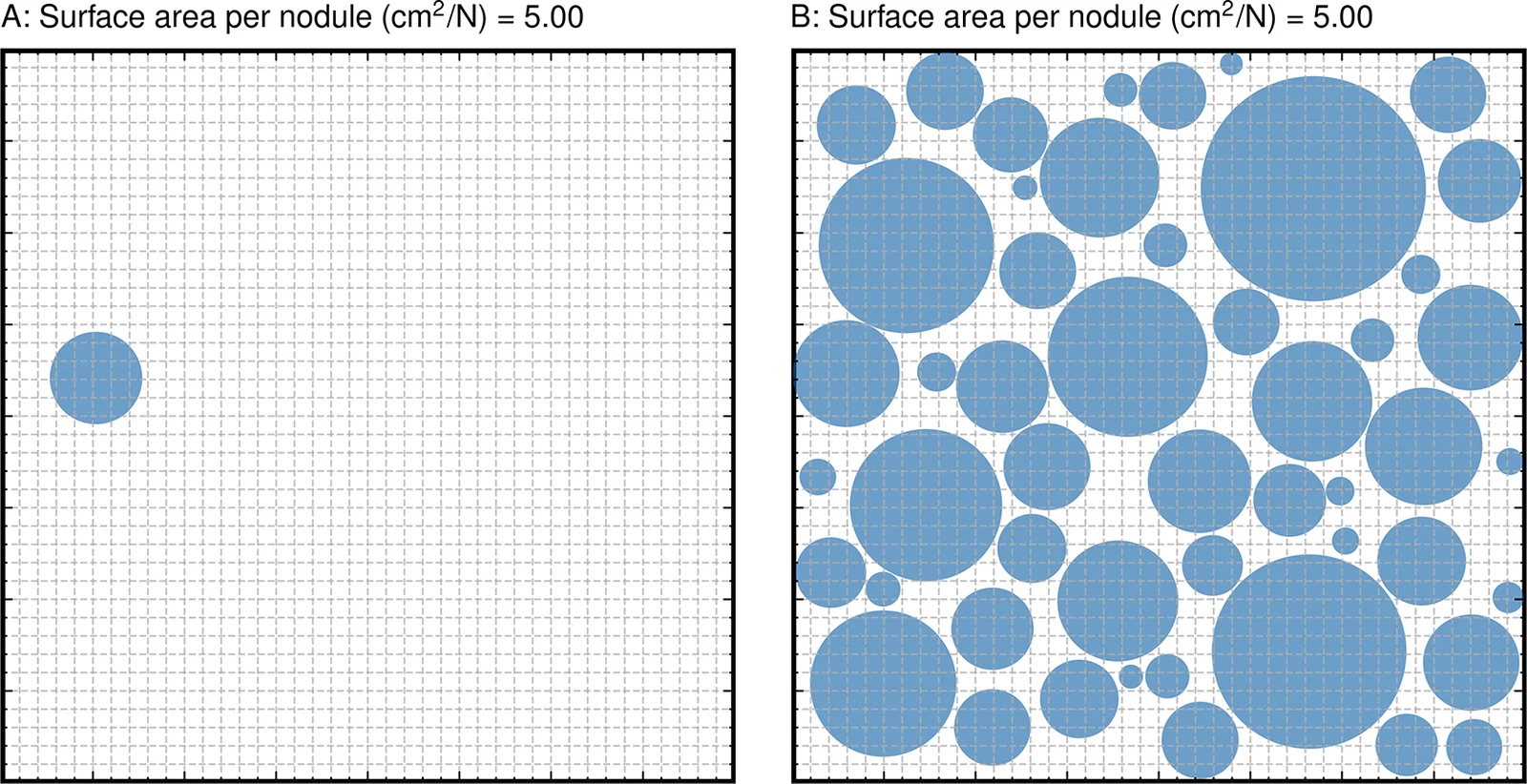
Schematic representations of nodules in a benthic chamber lander, viewed from above. These two chambers have the same average nodule surface area, but very different total surface areas and number of nodules.

In Sweetman et al. (2024), rising bottom water oxygen concentrations were observed in benthic chambers deployed on the seafloor of the CCZ. During the incubations, the chambers contained many things: sediment composed of various minerals and organic matter, sediment porewater, seawater, polymetallic nodules in some cases, and all of the various forms of life present in the water, sediment and nodules. In order to link the rising oxygen concentrations specifically to polymetallic nodules, the correlation between average nodule surface area (albeit a 2D, pseudo-surface area) and oxygen production rates was stated as a Spearman’s correlation coefficient with *⍴* = 0.664, *p* = 0.031, and the number of observations was 11 ( Figure 6A). The correlation was used to suggest that larger nodules results in more oxygen production. The average nodule surface area was obtained by dividing the total surface area of nodules in a chamber by the number of nodules in that chamber, it thus describes the mean average surface area of any one nodule in the chamber. Using the average surface area alone is problematic because any information about the size distribution, number of nodules or total amount of nodules is lost when this ratio is calculated. For any given value of a ratio, the magnitude of the numerator and denominator can be large or small, as long as the two are varied in tandem. In other words, a chamber with very few nodules could have the same average surface area as a chamber with a great many nodules ( Figure 7). The chambers in this study display considerable variation in both total nodule area and number of nodules ( Table 1). Consequently, the average surface area should be accompanied by some measure of nodule abundance — for example total surface area, or nodule weight. Therefore, correlating oxygen production against this average surface area alone is unlikely to demonstrate the relationship that was claimed in Sweetman et al. (2024), at least without a greater number of samples to better represent the natural range of nodule abundance. It is more likely that the low number of samples allowed a spurious correlation to emerge. We have demonstrated that including all the available data in the analysis results in a statistically insignificant correlation ( Figure 6B), confirming that the published correlation was spurious, and raises questions about the unexplained omission of chambers from the analysis of Sweetman et al. (2024).

Instead of average surface area, at least two alternative datasets are available that could be used to link polymetallic nodules to rising oxygen concentrations. The total surface area of nodules in each chamber could be used, however, there is no such correlation. The data presented in Sweetman et al. (2024) has a non-significant Spearman’s correlation coefficient of *⍴* = −0.136, p = 0.69 ( Figure 4A). After applying our segmentation method to these data, together with the three additional chambers, we also find no correlation ( Figure 4B). The weight of nodules collected from the chambers could have been used, which is a direct measurement of the size and quantity of nodules present in the chamber whilst also having a higher statistical power due to the greater number of samples (n = 17). Neither the weight of nodules in the upper layer, nor the total weight of nodules, nor the average weight of nodules show a correlation ( Figure 4C, D, Figure 5). The lack of correlation with average nodule weight is particularly revealing, since it represents a similar metric to the average surface area in that it describes the size of the nodules present, but is more robust because it uses directly obtained empirical data rather than a derived and somewhat problematic 2D pseudo-surface area. Collectively, these results clearly demonstrate that oxygen production is not related to the size or number of nodules in the chamber, and therefore they do not support a relationship between polymetallic nodules and the reported rises in oxygen concentration.

It is highly pertinent to the discussion to note that chambers collected in the western CCZ on cruise KM1808 of the R/V Kilo Moana did not contain manganese nodules. ^14, 15^ Yet, deployments AKS254, AKS257 and AKS261 from this expedition recorded increases in oxygen concentration of a similar magnitude to the experiments that included polymetallic nodules ( Table 1). These results preclude the possibility that oxygen production in those chambers was due to the presence of polymetallic nodules, because no nodules were present. Data from these chambers were used to support the hypothesis that oxygen concentration increases were attributable to polymetallic nodules in Sweetman et al. (2024), whilst the absence of nodules in those chambers was not reported and the earlier studies ^14, 15^ that documented the absence of nodules were not cited. Furthermore, benthic chamber lander experiments, ex-situ incubations, nodule-only incubations and voltage measurements on nodule surfaces were conducted in 2021, 2022 and 2023, ^1^ when it was known that oxygen concentrations had previously risen without nodules present. It is unclear why such extensive effort was devoted to testing a hypothesis that had already been robustly rejected. Conversely, no effort was made to test the hypothesis that sediment without nodules could cause oxygen concentration increases, despite sediment being present in all of the chambers, including when nodules were absent. In addition, a control experiment that was conducted without nodules or sediment also recorded rising oxygen concentrations, and was also omitted from Sweetman et al. (2024). ^7^ This control experiment rules out the possibility for polymetallic nodules or the sediment or other materials, such as small particles of manganese oxide or other mineral phases in the sediment, from causing the reported rises in oxygen concentration.

The only remaining piece of evidence that links polymetallic nodules to rising oxygen concentrations is a single ex-situ incubation performed with one polymetallic nodule immersed in seawater. ^1^ However, there is little detail about this experiment, including how the nodule was stored prior to the incubation commencing, or the composition of the gas that was produced during the incubation. A simple analysis of the gas would determine if it was atmospheric in composition, which would be consistent with the release of trapped air rather than *in situ* oxygen production. We note that in the time since Sweetman et al. (2024) was published, no study has replicated this result, despite nodules being readily available at various institutions around the world. Such a finding—that polymetallic nodules can spontaneously produce oxygen when submerged in seawater—would be extraordinary, and likely violate the established laws of thermodynamics. ^7, 9^ Furthermore, this experiment is at odds with the data from the western CCZ ^14, 15^ where oxygen concentrations rose without any polymetallic nodules present at all, and the control experiment conducted without nodules or sediment. ^7^

## Conclusion

Re-analysis of the data presented by Sweetman et al. (2024), together with additional datasets, demonstrates no relationship between polymetallic nodules size or abundance and rising oxygen concentrations measured in benthic chambers. Combined with the experiments conducted in the western CCZ that recorded rising oxygen concentration in the absence of nodules, ^1, 14, 15^ the nodule-free control experiment, ^7, 8^ and the prior work showing only oxygen consumption in nodule-rich areas, ^2– 6^ these findings collectively reject the hypothesis that polymetallic nodules caused the observed oxygen concentration increases. There is now a substantial body of evidence ^7– 13, 20^ that challenges the observations and claims reported in Sweetman et al. (2024). Furthermore, examination of the experimental timeline suggests that the authors should have been aware that their primary hypothesis was untenable years before further experiments were conducted.

## Ethical considerations

Not applicable.

## Data Availability

Chamber photographs, oxygen data, optode data, nodule weight data, offshore logs and other metadata are made available on Zenodo: https://zenodo.org/records/19707357 ^21^ under the terms of the Creative Commons Attribution 4.0 International. The python code use to generate the results in this study is available on GitHub *:* https://github.com/apwebber/SegmentAKS with an archive of version 0.1.2 that was used here on Zenodo: https://zenodo.org/records/19848199 ^22^ under the terms of the MIT License. Not applicable.
