# Supplementary material for: No link between polymetallic nodules and anomalous oxygen concentration increases in benthic experiments from the Clarion Clipperton Zone
Source: F1000Res. 2026 Sep 18;15:729. Originally published 2026 May 14. [Version 2] doi: 10.12688/f1000research.181486.2 (PMC13560710; doi:10.12688/f1000research.181486.2)
Supplement: Peer review history

# Reviewer report: Reviewer response for version 2

**DOI:** 10.5256/f1000research.209788.r504678

**Published:** 2026-09-23

**Authors:** Sabine Kasten

**Affiliations:** 1 Alfred Wegener Institute Helmholtz Centre for Polar and Marine Research, Bremerhaven, Germany

I have examined the manuscript submitted by Webber et al. as well as the 4 reviews already available for this submission. I agree with many of the comments and arguments given in these 4 evaluations – in particular those given by Walter Geibert and Clare Reimers. I therefore do not want to repeat all of them here in detail – but rather mention and echo selected issues and aspects that are most important from my point of view and add a few additional comments.

 The manuscript submitted by Webber et al. has re-examined original data presented by Sweetman et al. (2024) and included three additional sites that were not part of the initial statistical analyses by Sweetman et al. The peer-reviewed article by Sweetman et al. (2024) that was published in Nature Geoscience reported a positive correlation between nodule surface area - determined based on an image analysis approach – and the increase in oxygen concentrations in benthic chamber

 experiments/deployments/incubations. They hypothesized that seawater electrolysis may contribute to this dark oxygen production (DOP). The article by Sweetman et al. (2024) has received a lot of attention in the scientific community and has provoked a very controversial debate. So any follow-up studies to re-evaluate these findings and in particular to advance our understanding of this observation are highly welcome and of profound scientific interest.

 Based on their re-evaluation of the data of Sweetman et al. (2024), Webber et al. report that they have not found a significant positive correlation between the nodule surface area determined based on an image analysis approach and the amount of oxygen produced in the benthic chamber experiments. They do not doubt or question the dark production of oxygen in these abyssal environments. However, unfortunately, they also do not further discuss potential processes or conditions most likely driving the increase in oxygen concentrations observed in the lander experiments and thus do not significantly contribute to advancing our understanding of this „observation“. Together with the fact that all 6 authors are (one was) employed at „The Metals Company“, this submission – as already stated by Clare Reimers – gives the strong impression that the prime objective is to prove the results and conclusions of Sweetman et al. (2024) wrong by demonstrating that there is no relationship between the abundance or surface area of polymetallic nodules and the production of oxygen in this dark deep-sea setting. This is not surprising as „The Metals Company“ of course has a strong (economic) interest that any further scientific observations adding to the wealth of findings that argue against potential deep-sea mining, are rather refuted or at least weakened.

 The fact that the authors state that oxygen is obviously also produced in sediments where no (large or visible) polymetallic concretions are found at the sediment surface makes this observation even more interesting and demonstrates - as already stated by Sweetman et al. - that the process/es potentially generating oxygen in these abyssal environments is/are far from being understood. This may be due to the fact that the approaches applied to determine the surface area of the nodules (and other polymetallic precipitates) present at or within the surface sediments are either inappropriate and/or that other or additional factors and conditions determining the rate of this process are not taken under consideration. As already pointed out by Clare Reimers, I am also very sceptical about the way the surface area of the Mn nodules located at the seafloor has been determined – both by Sweetman et al. (2024) and by Webber et al. – namely by means of an image analyis approach. This is certainly not the appropriate approach to determine the specific surface area of these mineral precipitates. Based on the wealth of literature on shape, size, surface texture and mineralogy of Mn nodules as well as more than 15 years of own research and numerous cruises in the area of the CCZ, nodules in the CCZ were show to display a broad spectrum of surface structure/texture (e.g. smooth to botryoidal), shape, size and mineralogy. The approach chosen here is definitely not capable to take this natural variability into account. Moreover, the (undisturbed) surface sediments of the CCZ are known to typically show high enrichments of authigenic Mn precipitates that appear finely dispersed within the sediment as well as abundant micro-nodules. To me it appears plausible that besides the abundance of large nodules the high specific surface area of these fine-grained Mn prepicitates (invisible to image analysis software) together with the mineralogy of the precipitates will determine the overall/total specific surface area and control the (bulk) reactivity of the polymetallic precipitates present.

 These considerations may underpin the broadly accepted insight that our understanding of conditions prevailing and processes acting in these remote abyssal seafloor environments is still very limited. However, the knowledge collected over more than 7 or 8 decades of research in the CCZ is sufficient to know with a high degree of certainty that any future mining activities removing nodules and heavily disturbing, removing and suspending the surface sediments will heavily impact and destroy these unique abyssal environments and ecosystems. Polymetallic nodules found in the CCZ and in other deep-sea environments have been shown to grow over time scales of several millions of years. So, targeting these precipitates during any potential future mining activities will irretrievably remove them from the deep-sea environment in conjunction with the unique ecosystems that rely on polymetallic nodules as hard grounds/substrate and the sessile organisms attached to these. Also the impacts of disturbance, removal and resuspension of the surface sediments – which typically goes along with any know (potential) technique collecting nodules from the ocean floor – will be profound and manifold. The sediments in the CCZ accumulate at extremely low rates - typically well below 0.5 cm/1000 years. So any natural recovery of the surface sediment habitat will take several tens of thousands of years. In addition, the organic carbon contents in these surface sediments are surprisingly high for the prevailing water depths – mostly due to the extended mid-depth oxygen minimum zone found in the equatorial Pacific Ocean. As a consequence, suspending these organic-carbon containing fine-grained sediments would enhance the mineralisation/oxidation of organic carbon in the water column, releasing CO _2_ as a consequence and consuming oxygen in the lower water column. At the same time the intense re-suspension induced by any mining operation will significantly weaken the natural longterm carbon storage capacity of theses surface sediments.

 So, to conclude I find it very interesting to see that the re-evaluation of the data suggests that oxygen concentrations do not only rise in benthic chamber incubations of seafloor areas covered by large polymetallic nodules but also in seafloor habitats barren of large/visible nodules. The findings of both Sweetman et al. (2024) and Webber et al. do not question dark oxygen production in these settings. However, as already mentioned above the work submitted by Webber et al. does not discuss, present or offer any novel scientific insight or aspects to advance the understanding of the observed increase in oxygen concentrations in the benthic chamber experiments. Besides, the numerous formal aspects (e.g. interpretation of the results of the statistical analysis, referencing) that are for example outlined in great detail in the review of Walter Geibert, I think there is a need to include and carefully discuss additional relevant scientific aspects, conditions and parameters that might be capable of explaining their observations, before I can approve this submission.

Is the work clearly and accurately presented and does it cite the current literature?

No

If applicable, is the statistical analysis and its interpretation appropriate?

Partly

Are all the source data underlying the results available to ensure full reproducibility?

Yes

Is the study design appropriate and is the work technically sound?

Partly

Are the conclusions drawn adequately supported by the results?

Partly

Are sufficient details of methods and analysis provided to allow replication by others?

Yes

Reviewer Expertise:

sediment geochemistry and biogeochemistry, early diagenesis in marine sediments, polymetallic precipitates and other authigenic marine minerals, carbon cycle

I confirm that I have read this submission and believe that I have an appropriate level of expertise to state that I do not consider it to be of an acceptable scientific standard, for reasons outlined above.

# Reviewer report: Reviewer response for version 2

**DOI:** 10.5256/f1000research.209788.r522405

**Published:** 2026-09-22

**Authors:** Klaas Meyn

**Affiliations:** 1 Federal Institute for Geosciences and Natural Resources, Hannover, Germany

All comments addressed and explanations added as requested, figure changed according to suggestions. Good scientific work.

Is the work clearly and accurately presented and does it cite the current literature?

Yes

If applicable, is the statistical analysis and its interpretation appropriate?

Yes

Are all the source data underlying the results available to ensure full reproducibility?

Yes

Is the study design appropriate and is the work technically sound?

Yes

Are the conclusions drawn adequately supported by the results?

Yes

Are sufficient details of methods and analysis provided to allow replication by others?

Yes

Reviewer Expertise:

Deep-sea biology, benthic ecology, hydrothermal vent fields, megafauna

I confirm that I have read this submission and believe that I have an appropriate level of expertise to confirm that it is of an acceptable scientific standard.

# Reviewer report: Reviewer response for version 1

**DOI:** 10.5256/f1000research.200327.r504676

**Published:** 2026-08-25

**Authors:** Clare E. Reimers

**Affiliations:** 1 College of Earth, Ocean, and Atmospheric Sciences, Oregon State University, Corvallis, Oregon, USA

Webber *et al. *in their manuscript in open peer review “ *Anomalous oxygen concentration increases in benthic experiments from the Clarion Clipperton Zone are not related to polymetallic nodules*” re-examine select data, analyses and conclusions of a controversial 2024 *Nature Geoscience* publication by Sweetman *et al.* that attributes increases in dissolved oxygen in benthic chambers placed over deep-sea sediments to “dark oxygen production” arising from seawater electrolysis driven by natural processes. Webber *et al.* do not challenge the finding of dark oxygen production (DOP) itself but rather a minor hypothesis that this production is related to the average planar surface area of ferromanganese nodules observed in multiple chamber experiments made at water depths >4200m.

 Their approach is to redo surface area measurements based on photographs of the recovered substrates in benthic chambers and to redo correlation analyses between these average “2D pseudo surface areas” and DOP, adding measurements from three chambers rejected by Sweetman *et a*l., as well as measures of total nodule area and weight. The reanalysis findings are more robust statistically than original assessments, and the conclusion is that the DOP rates derived from optode time-series measurements in chambers (and supported by Winkler measurements on water samples) did not have any significant correlations with the average size, or total number, or weights of nodules in the chambers. They also tabulate and emphasize three other chamber experiments from the western Clarion Clipperton Zone where DOP was observed at sites with no nodules.

 The underlying tactic of Webber *et al.* is to reject any connection between DOP and polymetallic nodules by showing the published area analyses were correct, but the data treatment was biased and incomplete. A weakness is no consideration is given to published work showing that nodules have extremely high specific surface areas when measured by N _2_ gas adsorption (see e.g., https://doi.org/10.3390/min13111431). Such measures would be more meaningful than average 2D area for assessing evidence for a role of nodule surfaces and voltage gradients in water electrolysis.

 The paper’s conclusion harshly states that Sweetman et al. should have been aware their primary hypothesis was “untenable”. Rather than making this judgement, a more constructive approach would have been original work designed to substantiate if DOP is a real phenomenon rather than a methodological artifact. The cited rebuttal preprint by Tengberg et al. raises several benthic chamber methodological concerns to which I would add possible electrolysis driven by unintended electrical paths across materials in the chambers.

 The jury is still out on the causes of the DOP measurements reported by Sweetman *et al*. (2024). What is without doubt is that the influence of ferromanganese nodules on abyssal ocean ecosystems is far from understood and has evolved over millions of years in an environment with little disturbance

Is the work clearly and accurately presented and does it cite the current literature?

Partly

If applicable, is the statistical analysis and its interpretation appropriate?

Yes

Are all the source data underlying the results available to ensure full reproducibility?

Yes

Is the study design appropriate and is the work technically sound?

Yes

Are the conclusions drawn adequately supported by the results?

Partly

Are sufficient details of methods and analysis provided to allow replication by others?

Yes

Reviewer Expertise:

Ocean science, marine technology  and benthic biogeochemistry.

I confirm that I have read this submission and believe that I have an appropriate level of expertise to confirm that it is of an acceptable scientific standard, however I have significant reservations, as outlined above.

## References

[1] : Hydrogenetic and Diagenetic Controls on the Specific Surface Area of Polymetallic Nodules in Deep Ocean Basins. *Minerals* .2023;13(11) : 10.3390/min13111431 10.3390/min13111431

# Response

**Published:** 2026-09-01

**Authors:** Alexander Webber

**Affiliations:** Environmental, The Metals Company, Vancouver, BC, Canada

**Reviewer:**

 Webber *et al. *in their manuscript in open peer review “ *Anomalous oxygen concentration increases in benthic experiments from the Clarion Clipperton Zone are not related to polymetallic nodules*” re-examine select data, analyses and conclusions of a controversial 2024 *Nature Geoscience* publication by Sweetman *et al.* that attributes increases in dissolved oxygen in benthic chambers placed over deep-sea sediments to “dark oxygen production” arising from seawater electrolysis driven by natural processes. Webber *et al.* do not challenge the finding of dark oxygen production (DOP) itself but rather a minor hypothesis that this production is related to the average planar surface area of ferromanganese nodules observed in multiple chamber experiments made at water depths >4200m.

*Reply: We thank Claire Reimers for their thoughtful review. We would add that we do not challenge the fundamental observation of rising oxygen concentrations in these experiments, as they were clearly measured by two different methods – but we do challenge that these have been successfully linked to a natural process and to polymetallic nodules in particular. We think that additions made in version 2 of the manuscript on the request of other reviewers will clarify this position.*

**Reviewer:**

 The underlying tactic of Webber *et al.* is to reject any connection between DOP and polymetallic nodules by showing the published area analyses were correct, but the data treatment was biased and incomplete. A weakness is no consideration is given to published work showing that nodules have extremely high specific surface areas when measured by N _2_ gas adsorption (see e.g., https://doi.org/10.3390/min13111431). Such measures would be more meaningful than average 2D area for assessing evidence for a role of nodule surfaces and voltage gradients in water electrolysis.

*Reply: We wholeheartedly agree that more robust measures of surface area would have been available to the authors of Sweetman et al. (2024). Unfortunately, we are now in a position of working with the available data, although this does not rule out the possibility of further, more rigorous, investigations in future. We will add N2 has adsorption as a possibility for future work.*

**Reviewer:**

 The paper’s conclusion harshly states that Sweetman et al. should have been aware their primary hypothesis was “untenable”. Rather than making this judgement, a more constructive approach would have been original work designed to substantiate if DOP is a real phenomenon rather than a methodological artifact. The cited rebuttal preprint by Tengberg et al. raises several benthic chamber methodological concerns to which I would add possible electrolysis driven by unintended electrical paths across materials in the chambers.

*Reply: In this work we originally wanted to refrain from speculating on the true cause of the rising oxygen, since such a discussion is not based on any data we present here. However, several reviewers including yourself have now asked for a bit more in this direction, and we have added a short discussion, which largely links to the other discussions that are available on this topic and*

 The jury is still out on the causes of the DOP measurements reported by Sweetman *et al*. (2024). What is without doubt is that the influence of ferromanganese nodules on abyssal ocean ecosystems is far from understood and has evolved over millions of years in an environment with little disturbance

- Is the work clearly and accurately presented and does it cite the current literature?

Partly

*Reply: We hope the inclusion of the N2 gas adsorption method as a possibility for future work will satisfy this point.*

- Is the study design appropriate and is the work technically sound?

Yes

- Are sufficient details of methods and analysis provided to allow replication by others?

Yes

- If applicable, is the statistical analysis and its interpretation appropriate?

Yes

- Are all the source data underlying the results available to ensure full reproducibility?

Yes

- Are the conclusions drawn adequately supported by the results?

Partly

*Reply: We hope that the additional discussion points will satisfy this point.*

# Reviewer report: Reviewer response for version 1

**DOI:** 10.5256/f1000research.200327.r504683

**Published:** 2026-08-04

**Authors:** Klaas Meyn

**Affiliations:** 1 Federal Institute for Geosciences and Natural Resources, Hannover, Germany

Webber et al. contribute to the ongoing debate on the hypotheses of potential "dark oxygen production" by re-analyzing existing data, adding new available data sets to their current analysis and present all methods deviating from those of Sweetman et al. (2024) in a transparent scientific way. They present several issues contradicting the results and interpretation of the Sweetman et al. (2024) publication.

 They do add valuable, additional information to the topic of DOP and address some key issues of the DOP production claim of the Sweetman et al. publication supported by re-analysis of the original data and new data that was omitted in the previous study.

 I only have some minor comments, in particular regarding the presentation of some of the resulting figures 1, 3, 4, 5 and 6.

 Figure 1:

 The k-means classes superimposed on the classes in the Quantized image are not clearly visible. Please consider to place the 4 classes next to the image or below for readability.

 Figure 3:

 which of the blue dots are referring to re-analyzed data point from Sweetman et al and which are new data point that were omitted in this study and presented here as new data points?

 Figure 4 (comments partially also valid for 5 and 6):

 1) Maybe it would be helpful to add the "no-correlation" lines in these figures to outline the not existing correlation? Just by comparing the four figures it appears to me that additional samples were added in B, C and D but the point distribution seems at least similar. To underline the not existing correlation it would be easier for the reader if these lines are added here, comparable to figure 3. Also it would be helpful to choose a different color for the new added data point that you analyzed in addition to the previous existing data.

 2) Why is the no-nodule data (nodule area/weight of 0 if I understand correct) not shown in this figure while it is discussed as example of oxygen increase in the absence of nodules? Maybe I am missing something but as this is part of your analysis why is it excluded here?

 Figures 5 and 6:

 Is this all new analyzed data or new and re-analyzed data? As I understand from the text, you re-analyzed and added new data for your re-evaluation of the Sweetman et al. results. Similar to my comment above, I suggest to use different colors for Sweetman et al re-analyzed data and the new data added in this study. In addition, the correlation line would make it easier to follow the correlation vs. no correlation discussion by visualizing it.

 Figure 7:

 As I am not familiar with benthic chamber work: Is it a normal procedure to calculate the surface area per nodule? What would be the advantage of it if there is any? Is is about the size of the nodules? It is a good figure to visualize the problem arising from this calculated proxy. This is just a question of personal interest and has been addressed adequately in your manuscript.

 These are all minor comments mainly suggesting some editing in presenting the data to make it easier for the reader to follow the discussion and the points the authors intend to present by showing the figures.

 As I am not an expert in benthic chamber work, biogeochemistry and oxygen consumption and its analyzing procedures, some of these comments might be misunderstandings and may only need clarification from the authors.

 Overall this study contributes to the discussion of DOP and add some minor but interesting new aspects to the numerous addressed issues with the original publication. It would have been nice to provide some explanations of why these oxygen optodes produce the observed initial measured oxygen increase. The authors indicate that this might be likely related to the chambers itself as an artefact, but remain speculative in its explanations. However, as pointed out by the authors, this was beyond the scope of the current analysis and they do nicely present their results and additional new data and contribute to the scientific debate.

Is the work clearly and accurately presented and does it cite the current literature?

Yes

If applicable, is the statistical analysis and its interpretation appropriate?

Yes

Are all the source data underlying the results available to ensure full reproducibility?

Yes

Is the study design appropriate and is the work technically sound?

Yes

Are the conclusions drawn adequately supported by the results?

Yes

Are sufficient details of methods and analysis provided to allow replication by others?

Yes

Reviewer Expertise:

Deep-sea biology, benthic ecology, hydrothermal vent fields, megafauna

I confirm that I have read this submission and believe that I have an appropriate level of expertise to confirm that it is of an acceptable scientific standard.

# Response

**Published:** 2026-09-01

**Authors:** Alexander Webber

**Affiliations:** Environmental, The Metals Company, Vancouver, BC, Canada

*We thank Klaas Meyn for their review. Our detailed responses are below and their suggestions will be incorporated into version 2 of the article.*

 Reviewer:

 Figure 1:

 The k-means classes superimposed on the classes in the Quantized image are not clearly visible. Please consider to place the 4 classes next to the image or below for readability.

*Response: We have now ensured the legend is legible.*

 Reviewer:

 Figure 3:

 which of the blue dots are referring to re-analyzed data point from Sweetman et al and which are new data point that were omitted in this study and presented here as new data points?

*Response: All the points represent samples which were published in both studies, plotted against each other to ensure we are not introducing some bias that would affect the interpretation. We will clarify the caption.*

 Reviewer:

 Figure 4 (comments partially also valid for 5 and 6):

 1) Maybe it would be helpful to add the "no-correlation" lines in these figures to outline the not existing correlation? Just by comparing the four figures it appears to me that additional samples were added in B, C and D but the point distribution seems at least similar. To underline the not existing correlation it would be easier for the reader if these lines are added here, comparable to figure 3. Also it would be helpful to choose a different color for the new added data point that you analyzed in addition to the previous existing data.

*Response: We provide the Spearman’s correlation coefficients for these data, following Sweetman et al. (2024), which is probably an appropriate metric to use since we are not sure of a linear relationship. This technique looks for any monotonic relationship between variables, equivalent to a Pearson’s correlation of the ranked data. Consequently, it would be inappropriate to put the regression line on these plots, which do not show the ranked data but the real values.*

 Reviewer:

 2) Why is the no-nodule data (nodule area/weight of 0 if I understand correct) not shown in this figure while it is discussed as example of oxygen increase in the absence of nodules? Maybe I am missing something but as this is part of your analysis why is it excluded here?

*Response: Although we considered adding these data to the plots, there are two problems. The calculation for the average surface area (total area/n) leads to an undefined surface area (0 divided by 0) and we would not be able to plot these. Secondly, the zero values for total area and total nodule weights are not based on empirical data but only what can be surmised from descriptions, and although it can be assumed they are zero we don’t feel we have enough confidence to plot them and include them in a correlation analysis, especially given that they stand far apart from the rest of the data and might have an undue influence on the correlation coefficient results. Therefore, we deal with them separately in the text.*

 Reviewer:

 Figures 5 and 6:

 Is this all new analyzed data or new and re-analyzed data? As I understand from the text, you re-analyzed and added new data for your re-evaluation of the Sweetman et al. results.

*Response: Although the total nodule weights (not broken down by weights at the surface and below the surface) were published in Sweetman et al. (2024) they were not interrogated. We plot that data (total nodule weight) as well as the weights of nodules at the surface of the chambers, which are equivalent to the nodules included in the surface area analysis.*

 Reviewer:

 Similar to my comment above, I suggest to use different colors for Sweetman et al re-analyzed data and the new data added in this study. In addition, the correlation line would make it easier to follow the correlation vs. no correlation discussion by visualizing it.

*Response: See comment on the correlation lines, which applies here as well. In terms of the colour of the data points, this is difficult because although Sweetman et al. (2024) published the total nodule weights, they did not interrogate the data in this way and so we feel that changing the colour would imply this relationship was already explored. We use a different colour for Fig 5A to denote that this was the relationship and correlation that was previously published.*

 Reviewer:

 Figure 7:

 As I am not familiar with benthic chamber work: Is it a normal procedure to calculate the surface area per nodule? What would be the advantage of it if there is any? Is is about the size of the nodules? It is a good figure to visualize the problem arising from this calculated proxy. This is just a question of personal interest and has been addressed adequately in your manuscript.

*Response: No, as far as we are aware this is not a common procedure and is somewhat ad-hoc given the low quality of the photographs and the fact that nodule weight data was available, which (as we state in the discussion) should have provided a much more robust measure of the quantity of nodules in the chambers. Image analysis of nodules is usually used when physical sampling would be impossible, for example by AUV over large areas. As reviewer #4 rightly points out, there are far more robust methods of obtaining the true surface area of the nodules.*

 Reviewer:

 These are all minor comments mainly suggesting some editing in presenting the data to make it easier for the reader to follow the discussion and the points the authors intend to present by showing the figures.

 As I am not an expert in benthic chamber work, biogeochemistry and oxygen consumption and its analyzing procedures, some of these comments might be misunderstandings and may only need clarification from the authors.

 Overall this study contributes to the discussion of DOP and add some minor but interesting new aspects to the numerous addressed issues with the original publication. It would have been nice to provide some explanations of why these oxygen optodes produce the observed initial measured oxygen increase. The authors indicate that this might be likely related to the chambers itself as an artefact, but remain speculative in its explanations. However, as pointed out by the authors, this was beyond the scope of the current analysis and they do nicely present their results and additional new data and contribute to the scientific debate.

*Response: We thank the reviewer for their interest, for the time taken to review and for the suggestions to clarify some points. Since reviewer #1 also asked for an opinion on the cause of the oxygen concentration increases, we will add a section on this in our revised manuscript.*

# Reviewer report: Reviewer response for version 1

**DOI:** 10.5256/f1000research.200327.r497204

**Published:** 2026-07-31

**Authors:** Walter Geibert

**Affiliations:** 1 Alfred Wegener Institute, Helmholtz Centre for Polar and Marine Research, Bremerhaven, Germany

**General comments**

 The manuscript “Anomalous oxygen concentration increases in benthic experiments from the Clarion Clipperton Zone are not related to polymetallic nodules” by Webber et al. (The Metals Company- competing interests are clearly stated) deals with an aspect of a previously published peer-reviewed article by Sweetman et al. (2024). In their manuscript, the authors re-analyze the original dataset used by Sweetman et al.. They investigate a correlation between variables that describe a polymetallic nodule occurrence vs. the reported oxygen concentration change. Webber et al. include three additional stations in their re-analysis, and find that the correlation for polymetallic nodule cover and oxygen is not present with the added data included. From this lack of correlation and from the oxygen increase found in a control station, they conclude that a causality between nodule presence and oxygen increase is impossible (see manuscript title).

 The new data are a relevant addition to the discussion around “dark oxygen production”, and a re-examination of the Sweetman et al. findings in many aspects is required and welcome. The pressure on science to be quick on this subject is very high, given the economic interest in exploiting polymetallic nodules. Still, maintaining established scientific standards and aiming for a careful, objective analysis is important, particularly given the potential scientific impact of the Sweetman et al study. In the current form, I see a need for improvements in organization, referencing, reasoning and phrasing for this manuscript to enter the peer reviewed literature.

 Regarding recommendation for approval, I would also consider that any statement made here that is exclusively based on preprints, not on own data and arguments, would become citable as peer-reviewed after acceptance.  

 I should add that I did not download the provided data package as it would have required downloading >2 GB of data as two zip files with unknown content, to be extracted on the local computer.

**Detailed Comments** (I have numbered the sentences for each paragraph in many instances because I found no line numbers to refer to.)

**Abstract – background**

 1 ^st^ sentence: The original paper addresses benthic chamber experiments, but also an ex-situ incubation.

**Abstract – results**

 1 ^st^ sentence: “…indicating that there is no link between the abundance or size of polymetallic nodules and rising oxygen concentrations.” If this is maintained as a conclusion, it should be moved to conclusions.

 3 ^rd^ sentence, “Furthermore…” This is a mix of results and conclusion. The result is the observation of increasing oxygen concentrations in the absence of visible nodules at the sediment surface.

**Abstract- conclusions**

 1 ^st^ sentence: The original paper also reports instances of decreasing oxygen concentrations. The complete absence of polymetallic nodules, which is essential for this conclusion, is not well supported by evidence (see comments below referring to the main text).

 Reasoning of the authors: The absence of a correlation is not the same as an absence of a relation. To give an example: Even if fifteen measurements of oxygen production/consumption vs. tree number in a forest might yield a wide range of positive and negative numbers (trees produce, but also consume oxygen, and some oxygen might be produced/consumed by moss), a poor correlation would not mean that trees are not related to oxygen production. (Vice versa, a correlation alone would also not prove the relation). Correlation is not a proof for causality, the absence of correlation is not a proof for the absence of causality.

**Introduction**

 3rd sentence “…the work has attracted criticism from the scientific community, detailing many methodological concerns and issues relating to experimental design, reporting and interpretation of the data. ^7-13^” While the number of citations would suggest strong support in the scientific literature, a detailed look (see commented references at the end of the review) reveals that none of these seven references is a peer-reviewed research article that substantiates the claim. One is a self-citation of an opinion article, most of them are unreviewed pre-prints, partly self-citations, and one is a peer-reviewed article, but only with a marginal reference to the subject that differs from the claim made (it refers to ocean chemistry 4 billion years ago and it mentions the paper in a different context and does not raise “methodological concerns and issues relating to experimental design, reporting and interpretation of the data”.

 5 ^th^ sentence: The sentence “However, Sweetman et al. (2024) failed...” is neither supported by references here, nor has it been demonstrated yet in the manuscript. This would be a statement for conclusions, if proven, not for an introduction. If this was the hypothesis that the authors intend to test in their reanalysis of Sweetman’s data, it might be placed at the end of the introduction and introduced as a hypothesis to be tested- ideally in a phrasing that is less personal, but rather focusing on the specific claim the authors wish to challenge. This also applies to the following statements (“fail…”, “fail…”, …), which are neither placed well in an introduction, nor are they phrased as neutrally as required for an objective scientific examination.

 The section starting from “Contrary to the finding in Sweetman et al. (2024), we find no significant correlation between oxygen concentration increases and average nodule surface area...” is a result, not an introduction.

 The entire following text:

 “We also find no significant correlation between total nodule surface area, nodule weight or average nodule weight and oxygen concentration increases. We therefore reject the claim that the magnitude of oxygen concentration increase is related to the size or abundance of polymetallic nodules present. These findings are consistent with the chambers and control experiment without nodules, which indicate that oxygen increased regardless of whether nodules were present or not. We therefore conclude that there is no link between polymetallic nodules and the observed rises in oxygen concentration in the benthic chamber [… ] These observations preclude the possibility that polymetallic nodules have any role in oxygen concentration increases in those experiments, and are incompatible with the stated correlation with nodule surface area.[…] respirometer experiments.”

 is a mixture of results and conclusions, and the conclusions remain to be supported at this stage. This should all go to results and conclusions, respectively, if it can be substantiated in the following text.

 Irrespective of the correct place in the text and the phrasing, in the sentence “Furthermore, Sweetman et al. (2024) fail to report that chambers from the western CCZ that recorded increasing oxygen did not contain any nodules,14,15”, the references are not fully supporting the statement. Reference 14 states actually “No nodules were collected as they were either not present or in form of manganese oxide granules”, which is not really the same as “no nodules were observed”. Reference 15 is a doctoral thesis that could not be retrieved online and no source was given. Given that the authors state themselves below that “It is highly pertinent to the discussion to note that chambers collected in the western CCZ on cruise KM1808 of the R/V Kilo Moana did not contain manganese nodules.14,15”, this is a substantial missing piece.

**Methods**

 Penultimate paragraph: “Although O2 flux and total O2 production in these chambers was not previously published, we have used available data ^17^…”. Reference 17 is a generic reference (“SciPy 1.0: fundamental algorithms for scientific computing in Python”) that does not provide available data as suggested by the text.

**Discussion, end of ^3rd^ paragraph**

 “Collectively, these results clearly demonstrate that oxygen production is not related to the size or number of nodules in the chamber” This is inaccurate. It demonstrates the absence of a correlation for these variables in this dataset. Not more, not less.

**Discussion, 3 ^rd^ sentence of 4 ^th^ paragraph**

 “These results preclude the possibility that oxygen production in those chambers was due to the presence of polymetallic nodules, because no nodules were present”. The available reference does not rule out the presence of manganese oxide granules. Even if the observed oxygen production was related to another component of the deep-sea floor, this would not touch the central part of the paper by Sweetman et al. (2024), since the scientific change of paradigm that led to its prominent publication is the production of dark oxygen at the seafloor, not the direct production of oxygen by polymetallic nodules.

**Discussion, last paragraph:**

 “…would be extraordinary, and likely violate the established laws of thermodynamics. ^7,9”^

 This is another citation of an opinion piece and an unreviewed preprint in support of a statement that is not directly related to the dataset shown. Thermodynamic conditions were not tested here. The system in a benthic chamber or within a polymetallic nodule is not homogenous, and it contains bound oxygen as well as energy. Stating that the observed oxygen release is thermodynamically impossible (which may or may not be the case) requires a sound scientific assessment, especially in such a central aspect.

**Conclusion, penultimate sentence**: “There is now a substantial body of evidence ^7-13, 20 ^that challenges the observations and claims reported in Sweetman et al. (2024)”. The only peer-reviewed research paper addressing this question in the list is reference 20.

**Conclusion, last sentence**: “Furthermore, examination of the experimental timeline suggests that the authors should have been aware that their primary hypothesis was untenable years before further experiments were conducted”. This sentence could be understood as an allegation, implying that data were intentionally misrepresented. The manuscript here explores one aspect of the Sweetman et al. paper, which is composed of various lines of evidence. While all of these lines of evidence can and should be tested with sound methods, the dataset shown here is no sufficient foundation to allow such far-reaching claims. It could be understood as a personal criticism, not a scientific argument, and should be removed. 

**Commented references with peer review status:**

 1.  Sweetman AK, Smith AJ, De Jonge DSW, et al.: Evidence of dark oxygen production at the abyssal seafloor. Nat. Geosci. 2024; 17: 1–16. Publisher Full Text

Published and peer-reviewed

 2.  Smith KL, Laver MB, Brown NO: Sediment community oxygen consumption and nutrient exchange in the central and eastern North Pacific. Limnol. Oceanogr. 1983; 28(5): 882–898. Publisher Full Text

Published and peer-reviewed

 3.  Khripounoff A, Caprais JC, Crassous P, et al.: Geochemical and biological recovery of the disturbed seafloor in polymetallic nodule fields of the Clipperton-Clarion Fracture Zone (CCFZ) at 5,000-m depth. Limnol. Oceanogr. 2006; 51(5): 2033–2041. Publisher Full Text

Published and peer-reviewed

 4.  Stratmann T, Voorsmit I, Gebruk A, et al.: Recovery of Holothuroidea population density, community composition, and respiration activity after a deep-sea disturbance experiment. Limnol. Oceanogr. 2018; 63(5): 2140–2153. Publisher Full Text

Published and peer-reviewed

 5.  Vonnahme TR, Molari M, Janssen F, et al.: Effects of a deep-sea mining experiment on seafloor microbial communities and functions after 26 years. Sci. Adv. 2020; 6: eaaz5922–eaaz5914. PubMed Abstract | Publisher Full Text | Free Full Text

Published and peer-reviewed

 6.  An SU, Baek JW, Kim SH, et al.: Regional differences in sediment oxygen uptake rates in polymetallic nodule and co-rich polymetallic crust mining areas of the Pacific Ocean. Deep-Sea Res. I Oceanogr. Res. Pap. 2024 May; 207: 104295. Publisher Full Text

Published and peer-reviewed

 7.  Downes P, Cuesta A, Denny A, et al.: Extraordinary claims require extraordinary evidence: Evaluating nodule-associated dark oxygen production. Front. Mar. Sci. 2025; 12. Publisher Full Text

Opinion article, self-citation

 8.  Downes P, Marsh L, Bento J, et al.: Contributions to the discussion of novel detection of dark oxygen production at the abyssal seafloor.2024. Reference Source

Unreviewed preprint, self-citation

 9.  Cuesta A, Jaspars M: Is abyssal dark oxygen production even possible at all?.2025 Mar 10 [cited 2025 Jun 10]. Reference Source

Unreviewed preprint

 10.  Nakamura K: Questioning Dark Oxygen Production in the Deep-sea Ferromanganese Nodule Field.2024 Oct 4 [cited 2025 Jun 10]. Reference Source

Unreviewed preprint

 11.  Tengberg A, Hall P, Kononets M: Rebuttal of Sweetman, A.K., Smith, A.J., de Jonge, D.S.W. et al. Evidence of dark oxygen production at the abyssal seafloor. Nat. Geosci. 2024 Oct 4 [cited 2025 Jun 10].Publisher Full Text Reference Source

Unreviewed preprint

 12.  Trellevik LK, Denny A, Svellingen W: Critical Review of the Article: “Evidence of Dark Oxygen Production at the Abyssal Seafloor” by Sweetman et al. in Nat. Geosci. 1–3 (2024). Geochemistry. 2024 [cited 2025 Dec 4]. Reference Source

Apparently, an unreviewed preprint, page not found

 13.  Trost K, Gennis RB, Allen JF, et al.: Oxygen reductase origin followed the great oxidation event and terminated the Lomagundi excursion. Biochim. Biophys. Acta Bioenerg. 2026 Apr 1; 1867(2): 149575. Publisher Full Text

Peer reviewed, but not supporting the statement

 14.  Cecchetto MM, Moser A, Smith CR, et al.: Abyssal seafloor response to fresh phytodetrital input in three areas of particular environmental interest (APEIs) in the western clarion-clipperton zone (CCZ). Deep Sea Res Part I Oceanogr Res Pap. 2023; 195: 1–12. Publisher Full Text

Peer reviewed, but not fully supporting the statement (see comments)

 15.  Cecchetto MM: Benthic Ecosystem Functioning of the Western Clarion-Clipperton Zone, Pacific Ocean, and the West Antarctic Peninsula [Doctor of Philosophy]. Edinburgh: Heriot-Watt University; 2022.

Link not found, publication status of statement unclear as part of a PhD thesis

 16.  Pedregosa F, Varoquaux G, Gramfort A, et al.: Scikit-learn: Machine learning in Python. J. Mach. Learn. Res. 2011; 12: 2825–2830.

Peer reviewed and published

               17.  Virtanen P, Gommers R, Oliphant TE, et al.: SciPy 1.0: fundamental algorithms for scientific computing in Python. Nat. Methods. 2020; 17(3): 261–272. PubMed Abstract | Publisher Full Text | Free Full Text

Peer-reviewed, one instance of citing not matching the statement

 18.  Vallat R: Pingouin: statistics in Python. J. Open Source Softw. 2018; 3(31): 1026. Publisher Full Text

Peer reviewed (open source software)

 19.  Hall P, Welsh AH: Limit theorems for the median deviation. Ann. Inst. Stat. Math. 1985 Dec 1; 37(1): 27–36. Publisher Full Text

Peer-reviewed reference (for statistical method)

 20.  Harrison D, Kolbusz JL, Bond T, et al.: Seafloor surficial sediment variability across the abyssal plains of the central and eastern pacific ocean. Front. Earth Sci. 2025 Apr 22; 13. Publisher Full Text

Peer reviewed, independent reference

 21.  Webber AP: Respirometer Data. Zenodo. 2026 [cited 2026 Apr 23]. Publisher Full Text Reference Source

Reference to data (1.9 GB zip file)

 22.  Webber AP: SegmentAKS. Zenodo. 2026 [cited 2026 Apr 25]. Publisher Full Text Reference Source

Reference to data (98 MB)

Is the work clearly and accurately presented and does it cite the current literature?

Partly

If applicable, is the statistical analysis and its interpretation appropriate?

Partly

Are all the source data underlying the results available to ensure full reproducibility?

Partly

Is the study design appropriate and is the work technically sound?

No

Are the conclusions drawn adequately supported by the results?

No

Are sufficient details of methods and analysis provided to allow replication by others?

Yes

Reviewer Expertise:

Marine geochemistry, uranium-series disequilibria, trace element cycles, natural radioactivity in polymetallic nodules

I confirm that I have read this submission and believe that I have an appropriate level of expertise to state that I do not consider it to be of an acceptable scientific standard, for reasons outlined above.

## References

[1] : Evidence of dark oxygen production at the abyssal seafloor. *Nature Geoscience* .2024;17(8) : 10.1038/s41561-024-01480-8737-739 10.1038/s41561-024-01480-8 PMC1306163741971976

[2] : Sediment community oxygen consumption and nutrient exchange in the central and eastern North Pacific1. *Limnology and Oceanography* .1983;28(5) : 10.4319/lo.1983.28.5.0882882-898 10.4319/lo.1983.28.5.0882

[3] : Geochemical and biological recovery of the disturbed seafloor in polymetallic nodule fields of the Clipperton-Clarion Fracture Zone (CCFZ) at 5,000-m depth. *Limnology and Oceanography* .2006;51(5) : 10.4319/lo.2006.51.5.20332033-2041 10.4319/lo.2006.51.5.2033

[4] : Recovery of Holothuroidea population density, community composition, and respiration activity after a deep‐sea disturbance experiment. *Limnology and Oceanography* .2018;63(5) : 10.1002/lno.109292140-2153 10.1002/lno.10929

[5] : Effects of a deep-sea mining experiment on seafloor microbial communities and functions after 26 years. *Science Advances* .2020;6(18) : 10.1126/sciadv.aaz5922 10.1126/sciadv.aaz5922 PMC719035532426478

[6] : Regional differences in sediment oxygen uptake rates in polymetallic nodule and co-rich polymetallic crust mining areas of the Pacific Ocean. *Deep Sea Research Part I: Oceanographic Research Papers* .2024;207: 10.1016/j.dsr.2024.104295 10.1016/j.dsr.2024.104295

[7] : Extraordinary claims require extraordinary evidence: evaluating nodule-associated dark oxygen production. *Frontiers in Marine Science* .2025;12: 10.3389/fmars.2025.1721853 10.3389/fmars.2025.1721853

[8] : Is abyssal dark oxygen production even possible at all?.2025; 10.31223/X5DT6P 10.31223/X5DT6P

[9] : Questioning Dark Oxygen Production in the Deep-sea Ferromanganese Nodule Field.2024; 10.31223/X5PH7F 10.31223/X5PH7F

[10] : Oxygen reductase origin followed the great oxidation event and terminated the Lomagundi excursion. *Biochimica et Biophysica Acta (BBA) - Bioenergetics* .2026;1867(2) : 10.1016/j.bbabio.2025.149575 10.1016/j.bbabio.2025.149575 PMC761869141338429

[11] : Abyssal seafloor response to fresh phytodetrital input in three areas of particular environmental interest (APEIs) in the western clarion-clipperton zone (CCZ). *Deep Sea Research Part I: Oceanographic Research Papers* .2023;195: 10.1016/j.dsr.2023.103970 10.1016/j.dsr.2023.103970

[12] : SciPy 1.0: fundamental algorithms for scientific computing in Python. *Nature Methods* .2020;17(3) : 10.1038/s41592-019-0686-2261-272 10.1038/s41592-019-0686-2 PMC705664432015543

[13] : Limit theorems for the median deviation. *Annals of the Institute of Statistical Mathematics* .1985;37(1) : 10.1007/BF0248107827-36 10.1007/BF02481078

[14] : Seafloor surficial sediment variability across the abyssal plains of the central and eastern pacific ocean. *Frontiers in Earth Science* .2025;13: 10.3389/feart.2025.1527469 10.3389/feart.2025.1527469

# Response

**Published:** 2026-09-01

**Authors:** Alexander Webber

**Affiliations:** Environmental, The Metals Company, Vancouver, BC, Canada

We thank Walter Geibert for their detailed and considered review. We will upload a revised manuscript in due course, addressing their comments as outlined below.

**Abstract – background**

 Reviewer: 1 ^st^ sentence: The original paper addresses benthic chamber experiments, but also an ex-situ incubation.

*Response: Here we specifically address the benthic chamber experiments, since there is clear contrary evidence in the form of incubations with no nodules and a control experiment with no nodules. However, we have added that the ex-situ experiment was used to support the attribution of rising oxygen to nodules. We also address the ex-situ incubation in the discussion.*

**Abstract – results**

 Reviewer: 1 ^st^ sentence: “…indicating that there is no link between the abundance or size of polymetallic nodules and rising oxygen concentrations.” If this is maintained as a conclusion, it should be moved to conclusions.

*Response: This has been moved*

 3 ^rd^ sentence, “Furthermore…” This is a mix of results and conclusion. The result is the observation of increasing oxygen concentrations in the absence of visible nodules at the sediment surface.

*Response: This has been moved and clarified.*

**Abstract- conclusions**

 Reviewer: 1 ^st^ sentence: The original paper also reports instances of decreasing oxygen concentrations. The complete absence of polymetallic nodules, which is essential for this conclusion, is not well supported by evidence (see comments below referring to the main text).

*Response: This is dealt with in further comments.*

 Reviewer: Reasoning of the authors: The absence of a correlation is not the same as an absence of a relation. To give an example: Even if fifteen measurements of oxygen production/consumption vs. tree number in a forest might yield a wide range of positive and negative numbers (trees produce, but also consume oxygen, and some oxygen might be produced/consumed by moss), a poor correlation would not mean that trees are not related to oxygen production. (Vice versa, a correlation alone would also not prove the relation). Correlation is not a proof for causality, the absence of correlation is not a proof for the absence of causality.

*Response: We have now avoided asserting absence of causality. Our position is that the evidence that supported the link between polymetallic nodules and rising oxygen is discredited because the correlation was spurious. And we further argue that the lack of correlation with other metrics – mainly nodule abundance – is problematic for the hypothesis since one would reasonably expect a correlation to emerge, and it hasn’t (with the available data).*

**Introduction**

 Reviewer: 3rd sentence “…the work has attracted criticism from the scientific community, detailing many methodological concerns and issues relating to experimental design, reporting and interpretation of the data. ^7-13^” While the number of citations would suggest strong support in the scientific literature, a detailed look (see commented references at the end of the review) reveals that none of these seven references is a peer-reviewed research article that substantiates the claim. One is a self-citation of an opinion article, most of them are unreviewed pre-prints, partly self-citations, and one is a peer-reviewed article, but only with a marginal reference to the subject that differs from the claim made (it refers to ocean chemistry 4 billion years ago and it mentions the paper in a different context and does not raise “methodological concerns and issues relating to experimental design, reporting and interpretation of the data”.

*Response: There are a few points to consider here, with the first being the most important and is relevant for later comments too.*

1. *The reference number 7, Downes et al. (2025), was published in Frontiers as an “Opinion” article. In Frontiers, Opinion articles are described as allowing “authors to contribute viewpoints on the interpretation of recent findings in any research area, value of the methods used, as well as weaknesses and strengths of scientific hypotheses.” and most importantly they are peer reviewed, the details of which you can see in the article sidebar at https://www.frontiersin.org/journals/marine-science/articles/10.3389/fmars.2025.1721853/full . Therefore, the arguments made in that article are already part of the peer-review literature. With that in mind, we have not made any statements in this article that will be introduced to the peer-reviewed literature beyond the substance of this article, which is stated as the objectives and laid out in the methods section.*
2. *The pre-print rebuttals are cited because they expand greatly on the arguments that were summarised in the peer-reviewed article Downes et al. (2025) and are written by qualified and well-regarded members of scientific community. Furthermore, F1000 explicitly allows the citation of pre-prints: “Preprints can be cited and listed in the reference list.” We suggest that not citing these would be a disservice to readers who will seek the most comprehensive analysis.*
3. *The reference Trost et al. (2026) specifically addresses one of the claims that were made in Sweetman et al. (2024). Sweetman et al (2024): “Future studies of DOP in the deep sea may also shed light on broader relationships between metal-oxide deposition, biological evolution and the **oxygenation of Earth**16,17.”, Trost et al. (2026): “There are also reports that ocean floor manganese nodules can synthesize O2 [18], but the nodules in question are formed and deposited with the help of O2, rendering any such contribution to pre-GOE O2 production unlikely at best.”. We therefore contend that this reference rightly points out a problem with Sweetman et al. (2024) – because the interpretation that this process might have been important for the oxygenation of the earth is problematic. However, we have changed the wording to clarify that this is about the suggested significance of the findings.*
4. *We also contend that self-citation is sometimes necessary to point readers to additional information and expanded arguments, and we don’t feel we have been excessive in this regard.*

Reviewer: 5 ^th^ sentence: The sentence “However, Sweetman et al. (2024) failed...” is neither supported by references here, nor has it been demonstrated yet in the manuscript. This would be a statement for conclusions, if proven, not for an introduction. If this was the hypothesis that the authors intend to test in their reanalysis of Sweetman’s data, it might be placed at the end of the introduction and introduced as a hypothesis to be tested- ideally in a phrasing that is less personal, but rather focusing on the specific claim the authors wish to challenge. This also applies to the following statements (“fail…”, “fail…”, …), which are neither placed well in an introduction, nor are they phrased as neutrally as required for an objective scientific examination.

*Response: We have removed language such as “fail…” as well as removing all the conclusions from the introduction section.*

 Reviewer: The section starting from “Contrary to the finding in Sweetman et al. (2024), we find no significant correlation between oxygen concentration increases and average nodule surface area...” is a result, not an introduction.

*Response: See above*

 Reviewer: The entire following text:

 “We also find no significant correlation between total nodule surface area, nodule weight or average nodule weight and oxygen concentration increases. We therefore reject the claim that the magnitude of oxygen concentration increase is related to the size or abundance of polymetallic nodules present. These findings are consistent with the chambers and control experiment without nodules, which indicate that oxygen increased regardless of whether nodules were present or not. We therefore conclude that there is no link between polymetallic nodules and the observed rises in oxygen concentration in the benthic chamber [… ] These observations preclude the possibility that polymetallic nodules have any role in oxygen concentration increases in those experiments, and are incompatible with the stated correlation with nodule surface area.[…] respirometer experiments.”

 is a mixture of results and conclusions, and the conclusions remain to be supported at this stage. This should all go to results and conclusions, respectively, if it can be substantiated in the following text.

*Response: As above*

 Irrespective of the correct place in the text and the phrasing, in the sentence “Furthermore, Sweetman et al. (2024) fail to report that chambers from the western CCZ that recorded increasing oxygen did not contain any nodules,14,15”, the references are not fully supporting the statement. Reference 14 states actually “No nodules were collected as they were either not present or in form of manganese oxide granules”, which is not really the same as “no nodules were observed”. Reference 15 is a doctoral thesis that could not be retrieved online and no source was given. Given that the authors state themselves below that “It is highly pertinent to the discussion to note that chambers collected in the western CCZ on cruise KM1808 of the R/V Kilo Moana did not contain manganese nodules.14,15”, this is a substantial missing piece.

*Response: Reference 15 is freely available from the Heriot-Watt University thesis repository, and we will add the url to the citation. We include both because they contain slightly different wordings, and the thesis provides more information on the sample handling and other details.*

*To answer the matter of the granules, we have expanded upon this in the discussion to answer your question and the questions of reviewer #1.*

**Methods**

 Reviewer: Penultimate paragraph: “Although O2 flux and total O2 production in these chambers was not previously published, we have used available data ^17^…”. Reference 17 is a generic reference (“SciPy 1.0: fundamental algorithms for scientific computing in Python”) that does not provide available data as suggested by the text.

*Reference 17 was erroneously given instead of reference 14. This will be corrected.*

**Discussion, end of ^3rd^ paragraph**

 Reviewer: “Collectively, these results clearly demonstrate that oxygen production is not related to the size or number of nodules in the chamber” This is inaccurate. It demonstrates the absence of a correlation for these variables in this dataset. Not more, not less.

*Response: We have changed the wording to accurately reflect that they do not support the hypothesis.*

**Discussion, 3 ^rd^ sentence of 4 ^th^ paragraph**

 Reviewer: “These results preclude the possibility that oxygen production in those chambers was due to the presence of polymetallic nodules, because no nodules were present”. The available reference does not rule out the presence of manganese oxide granules.

*Response: We have re-worked this section to give all the available information that we have about the sediment in these chambers. You are correct and the authors of references 14 and 15 make a clear distinction between “nodules” and “granules”. We now address this issue and the reasons why it is problematic for the hypothesis.*

 Reviewer: Even if the observed oxygen production was related to another component of the deep-sea floor, this would not touch the central part of the paper by Sweetman et al. (2024), since the scientific change of paradigm that led to its prominent publication is the production of dark oxygen at the seafloor, not the direct production of oxygen by polymetallic nodules.

*Response: Here, we only suggest that those oxygen increases have not been sufficiently attributed to polymetallic nodules in particular. That assertion is made in the abstract of Sweetman et al. (2024) and that specific relationship is interrogated throughout that manuscript.*

**Discussion, last paragraph:**

 “…would be extraordinary, and likely violate the established laws of thermodynamics. ^7,9”^

 This is another citation of an opinion piece and an unreviewed preprint in support of a statement that is not directly related to the dataset shown. Thermodynamic conditions were not tested here. The system in a benthic chamber or within a polymetallic nodule is not homogenous, and it contains bound oxygen as well as energy. Stating that the observed oxygen release is thermodynamically impossible (which may or may not be the case) requires a sound scientific assessment, especially in such a central aspect.

*Response: As stated above, reference 7 is peer reviewed and the thermodynamic problems are therefore established in the peer reviewed literature in that article. We do no further analysis on that aspect here. We agree that a sound scientific assessment is required, and therefore we reference the places that this assessment can be found.*

**Conclusion, penultimate sentence**: “There is now a substantial body of evidence ^7-13, 20 ^that challenges the observations and claims reported in Sweetman et al. (2024)”. The only peer-reviewed research paper addressing this question in the list is reference 20.

*Response: We are not sure if the reviewer is asking for a change here, but we will restate that reference 7 is peer reviewed and the other references expand greatly upon the arguments made in that article.*

**Conclusion, last sentence**: “Furthermore, examination of the experimental timeline suggests that the authors should have been aware that their primary hypothesis was untenable years before further experiments were conducted”. This sentence could be understood as an allegation, implying that data were intentionally misrepresented. The manuscript here explores one aspect of the Sweetman et al. paper, which is composed of various lines of evidence. While all of these lines of evidence can and should be tested with sound methods, the dataset shown here is no sufficient foundation to allow such far-reaching claims. It could be understood as a personal criticism, not a scientific argument, and should be removed. 

*Response: This is now removed.*

**Commented references with peer review status:**

 12.  Trellevik LK, Denny A, Svellingen W: Critical Review of the Article: “Evidence of Dark Oxygen Production at the Abyssal Seafloor” by Sweetman et al. in Nat. Geosci. 1–3 (2024). Geochemistry. 2024 [cited 2025 Dec 4]. Reference Source

Apparently, an unreviewed preprint, page not found

*Response: source updated*

 13.  Trost K, Gennis RB, Allen JF, et al.: Oxygen reductase origin followed the great oxidation event and terminated the Lomagundi excursion. Biochim. Biophys. Acta Bioenerg. 2026 Apr 1; 1867(2): 149575. Publisher Full Text

Peer reviewed, but not supporting the statement

*Response: addressed above*

 14.  Cecchetto MM, Moser A, Smith CR, et al.: Abyssal seafloor response to fresh phytodetrital input in three areas of particular environmental interest (APEIs) in the western clarion-clipperton zone (CCZ). Deep Sea Res Part I Oceanogr Res Pap. 2023; 195: 1–12. Publisher Full Text

Peer reviewed, but not fully supporting the statement (see comments)

*Response: addressed above*

 15.  Cecchetto MM: Benthic Ecosystem Functioning of the Western Clarion-Clipperton Zone, Pacific Ocean, and the West Antarctic Peninsula [Doctor of Philosophy]. Edinburgh: Heriot-Watt University; 2022.

Link not found, publication status of statement unclear as part of a PhD thesis

*Response: link added*

 17.  Virtanen P, Gommers R, Oliphant TE, et al.: SciPy 1.0: fundamental algorithms for scientific computing in Python. Nat. Methods. 2020; 17(3): 261–272. PubMed Abstract | Publisher Full Text | Free Full Text

Peer-reviewed, one instance of citing not matching the statement

*Response: citation issue fixed*

  

- Is the work clearly and accurately presented and does it cite the current literature?

Partly

*Response: Since the reviewer has not commented on the data presentation or lack of citation of relevant literature, we assume we have addressed their concerns in other aspects of the article.*

- Is the study design appropriate and is the work technically sound?

No

*Response:  Since the reviewer has not commented on the technical methodology or study design we assume we have addressed their concerns in other aspects of the article.*

- Are sufficient details of methods and analysis provided to allow replication by others?

Yes

- If applicable, is the statistical analysis and its interpretation appropriate?

Partly

*Response: We hope the reviewer will now find that the interpretation is appropriate.*

- Are all the source data underlying the results available to ensure full reproducibility?

Partly

*Response: We assume this is related to the issue with referencing the Cecchetto et al. (2023) data, and this is now corrected.*

- Are the conclusions drawn adequately supported by the results?

No

*Response: We hope that the reviewer will find the conclusions more balanced and technically sound.*

# Reviewer report: Reviewer response for version 1

**DOI:** 10.5256/f1000research.200327.r491404

**Published:** 2026-06-26

**Authors:** Huaiming Li

**Affiliations:** 1 Second Institute of Oceanography, Ministry of Natural Resources, Hangzhou, China

Sweetman et al. (2024) first proposed that "dark oxygen" can be generated on the deep-sea floor in association with polymetallic nodules. This is a highly novel scientific hypothesis that continues to attract significant attention.

 Personally, I believe this hypothesis will remain the subject of considerable debate for a long time, and our understanding of the deep sea requires the continued accumulation of extensive observational data. Building upon the work of Sweetman (2024), this manuscript presents supplementary analytical data and results related to nodule surface area, weight, and abundance.

 The results indicate that the increase in dissolved oxygen on the deep-sea floor is unrelated to these nodules. These findings are of great value for further exploration of the dark oxygen issue, and I recommend this paper for indexing.

 However, I have a question regarding the results of this manuscript and would like the authors to provide clarification in the text. Do the authors consider the observed increase in dissolved oxygen content on the deep-sea floor in Sweetman et al. (2024) to be valid? If the results are not valid, does this imply that the findings of Sweetman et al. (2024) are erroneous? Alternatively, if the results are valid, what is the actual cause of the elevated dissolved oxygen content on the deep-sea floor?

Is the work clearly and accurately presented and does it cite the current literature?

Yes

If applicable, is the statistical analysis and its interpretation appropriate?

Yes

Are all the source data underlying the results available to ensure full reproducibility?

Yes

Is the study design appropriate and is the work technically sound?

Yes

Are the conclusions drawn adequately supported by the results?

Yes

Are sufficient details of methods and analysis provided to allow replication by others?

Yes

Reviewer Expertise:

Deep-sea Mineral Resource Exploration, Evaluation, and Ore-forming Model Research

I confirm that I have read this submission and believe that I have an appropriate level of expertise to confirm that it is of an acceptable scientific standard.

# Response

**Published:** 2026-07-08

**Authors:** Alexander Webber

**Affiliations:** Environmental, The Metals Company, Vancouver, BC, Canada

Dear Dr Li,

 We thank you for this considered review and for recommending the paper for indexing.

 In response to your questions, the growing body of evidence and reanalysis of the results presented in Sweetman et al. strongly suggest that the reported "dark oxygen" process does not represent a valid natural process of oxygen production. Rather, the signal is more plausibly explained as an experimental artefact, with the oxygen signal originating from the equipment itself rather than the nodules. This is covered in detail in the references we cite in the Conclusion section, including Tengberg et al. (2024), Cuesta and Jaspars (2025), Trellevik et al. (2024), Downes et al. (2024), Nakamura (2024), and the synthesis presented in Downes et al. (2025).

 We hope this clarification addresses your questions. We will revise the main text to make clearer how this contribution complements the existing body of evidence and further supports the experimental artefact explanation.

 Thank you again for your constructive review.
